# IRT and GPR Techniques for Moisture Detection and Characterisation in Buildings

**DOI:** 10.3390/s20226421

**Published:** 2020-11-10

**Authors:** Iván Garrido, Mercedes Solla, Susana Lagüela, Norberto Fernández

**Affiliations:** 1 GeoTECH Group, CINTECX, Universidade de Vigo, 36310 Vigo, Spain; ivgarrido@uvigo.es (I.G.); merchisolla@uvigo.es (M.S.); 2 Defense University Center, Spanish Naval Academy, Plaza de España s/n, 36900 Marín, Spain; norberto@cud.uvigo.es; 3 Department of Cartographic and Terrain Engineering, University of Salamanca, Calle Hornos Caleros, 50, 05003 Ávila, Spain

**Keywords:** infrared thermography, ground-penetrating radar, complementarity, moisture severity classification, building indoors

## Abstract

The integrity, comfort, and energy demand of a building can be negatively affected by the presence of moisture in its walls. Therefore, it is essential to identify and characterise this building pathology with the most appropriate technologies to perform the required prevention and maintenance tasks. This paper proposes the joint application of InfraRed Thermography (IRT) and Ground-Penetrating Radar (GPR) for the detection and classification of moisture in interior walls of a building according to its severity level. The IRT method is based on the study of the temperature distribution of the thermal images acquired without an application of artificial thermal excitation for the detection of superficial moisture (less than 15 mm deep in plaster with passive IRT). Additionally, in order to characterise the level of moisture severity, the Evaporative Thermal Index (ETI) was obtained for each of the moisture areas. As for GPR, with measuring capacity from 10 mm up to 30 cm depth with a 2300 MHz antenna, several algorithms were developed based on the amplitude and spectrum of the received signals for the detection and classification of moisture through the inner layers of the wall. In this work, the complementarity of both methods has proven to be an effective approach to investigate both superficial and internal moisture and their severity. Specifically, IRT allowed estimating superficial water movement, whereas GPR allowed detecting points of internal water accumulation. Thus, through the combination of both techniques, it was possible to provide an interpretation of the water displacement from the exterior surface to the interior surface of the wall, and to give a relative depth of water inside the wall. Therefore, it was concluded that more information and greater reliability can be gained by using complementary IRT-GPR, showing the benefits of combining both techniques in the building sector.

## 1. Introduction

The negative effects of moisture on building envelopes are well known thanks to previous studies found in the literature. These include: (i) loss of material in the affected structure due to changes in water volume when changing the physical phase and/or when chemical substances contained in water (e.g., salt) react with the affected material (e.g., plaster), (ii) worsening of user’s health and comfort due to the growth of mould in non-nutritive moist materials with traces of organic matter contamination, and (iii) increase in energy consumption due to the lower thermal resistance of moist materials compared to dry materials leading to a lower degree of insulation of a building. These negative effects can (i) lead to catastrophic consequences for the integrity of the building, (ii) cause illness and discomfort to users, and (iii) lead to a high level of energy waste, resulting in both increased economic costs and pollution.

In recent years, Non-Destructive Testing (NDT) techniques have become the most appropriate tools for the evaluation of any infrastructure, regardless of its nature. According to the ISO standard definitions [1], a technique is classified as NDT if it is an analysis technique used to evaluate the properties of a material, component, or system without causing damage. Then, an NDT technique is a specific way of using an NDT method, and an NDT method is a discipline that applies a physical principle in NDT [2]. According to ASTM E1316-20 [3], the list of NDT methods includes, among others, acoustic emission, electromagnetic testing, gamma- and X-radiology, leak testing, liquid penetrant testing, magnetic particle testing, neutron radiology and gauging, ultrasonic testing, etc. In this way, the intrusive nature of the destructive techniques and the alterations they cause in the integrity of the structure are avoided with NDT techniques [4], in addition to gaining in objectivity and speed in results generation [5]. 

There is a wide variety of published papers that use NDT techniques for the detection and characterisation of moisture severity in buildings, both superficial and internal. Recently, Rymarczyk et al. [6] employed electrical tomography in the spatial analysis of moisture in porous building materials (such as bricks and cement) of various thicknesses. Using gamma-rays and X-rays, Guimarães et al. [7] and Ren et al. [8] presented an experimental campaign for the critical analysis of water absorption in red brick samples, and they determined the moisture diffusivity of calcium silicate, ceramic brick, and lime mortar. Regarding dielectric and microwave meters, Hoła and Sadowski [9] proposed a method for identifying moisture content in saline brick walls. Additionally, Hoła et al. [10] analysed moisture content in thick masonry walls using a dielectric meter, and Teng et al. [11] presented a novel sensing technique for testing and evaluating moisture content and deterioration in concrete using a smart antenna and based on microwave frequencies. Another interesting NDT technique is laser scanning: recent studies published by Lerones et al. [12] and Suchocki et al. [13] describe a procedure to automatically show where moisture appears in a historic building and assess moisture saturation and movement in building materials, respectively. There is also recent research, such as that of Valero et al. [14], applying InfraRed Thermography (IRT) and electrical resistance measurement to the study of façades of historic buildings for in situ assessment of superficial moisture.

This work presents the combined use of two complementary NDT techniques for the detection and characterisation of moisture areas barely visible to the human eye on interior walls of a building: IRT is applied to the detection of superficial moisture (up to 1.5 cm depth in plaster using active IRT [15], lower depth with passive IRT), and Ground-Penetrating Radar (GPR) is used for internal moisture detection (deeper measurement than IRT, comprising the entire thickness of the wall with a maximum resolution of 1 cm). In this study, passive IRT was used because the aim is to develop a methodology with in situ applicability to allow the analysis of elements at every scale (walls and ceilings, in both indoor and outdoor scenarios). Conversely, active IRT requires a high thermal excitation of the element under study, which implies controlled ambient conditions and a robust energy supply, which are both difficult to obtain in in situ studies and for big scale scenarios. 

According to the ISO standard definitions [16], IRT is a technique that performs the imaging of the infrared (thermal) radiation emitted by objects. The infrared spectrum presents wavelengths ranging from 0.4 to 1000 µm [17], and it is divided in four sub-bands: Near InfraRed (NIR), Short-Wave InfraRed (SWIR), Mid-Wave InfraRed (MWIR) and Long-Wave or Thermal InfraRed (LWIR or TIR). IRT measures the radiation emitted by any object at a temperature over 0 K by means of a group of sensors sensitive to the TIR with wavelengths/frequencies ranging from 7 to 14 µm/from 2.1 × 10^4^ to 4.3 × 10^4^ GHz. Under ambient conditions, and without any thermal excitation applied by artificial sources to the object under study, TIR is the appropriate band for the measurement of temperatures through the thermal radiation. Regarding the distribution, the sensors are usually placed in a matrix shape and this matrix is typically installed inside a camera, known as InfraRed (IR) camera [18]. Then, the spatial distribution of the temperature values of the object under study is obtained by the Stefan–Boltzmann law using the measured radiation as input [17], resulting in a thermal image, or several thermal images in case of monitoring. It should be noted that the following correction factors must be applied if accurate temperature values are required [18]: (i) emissivity, (ii) reflected temperature, and (iii) atmospheric attenuation.

According to ASTM D6432-11 [19], GPR uses high-frequency-pulsed ElectroMagnetic (EM) waves (from 10 to 3000 MHz, belonging to the radio spectrum) to acquire information from the interior of objects. The EM waves are propagated through the object under study from a transmitting antenna. A fraction of the waves is reflected to a receiving antenna, when the EM waves pass through an interface between two media with different dielectric properties (mainly dielectric constant or permittivity). The amplitude value (or strength) of the reflected pulses is proportional to the dielectric contrast value between two media. The receiving antenna can be installed either in the same antenna box as the transmitting antenna or in a separate box [20]. It should be noted that an XZ image (typically called radargram) is obtained as a result of the GPR analysis. The values of the radargram represent the amplitude values of the different reflected pulses, which are measured as the transmitting antenna moves along the surface under study [21]. Thus, the X axis represents the distance (meters) of each trace (position of each pulse received) and the Z axis represents the two-way travel time of the radar pulses through the media. In addition to the amplitude of reflection, other attributes of the received GPR signal can be studied such as the amplitude spectrum, the velocity of propagation, and the dielectric constant of the propagation medium [20].

### 1.1. Applications of IRT and GPR in Building Moisture Analysis

Regarding IRT, surveys can be found in the literature, such as Kylili et al. [22] and Kirmtat and Krejcar [23], that analyse moisture in buildings through its different thermal characteristics with respect to the unaltered surroundings. Moreover, the work by Balaras and Argiriou [24] is one of the first papers to review the main areas of use of IRT in building diagnostics, including moisture damage identification, with an emphasis on how IRT is applied to support office building audits. A more recent review in the applications of IRT to building energy audits is the paper from Lucchi [25]. Lucchi states that moisture evaluation can be performed based on the analysis of (i) relative values of pixels of the thermal images to map moisture (qualitative analysis) or (ii) absolute values of the pixels of the thermal images to measure moisture content (quantitative analysis). Furthermore, the previous work states that water leaks, penetrative, and rising water through the building elements are the main causes of moisture in buildings, and as such, these must be taken into account on energy audits, prevention, and restoration works, among others. Another recent and comprehensive review on IRT is the paper by Nardi et al. [26], who analyse the use of IRT for the quantification of thermal energy losses through the building envelope. According to this latest review, moisture content is the most important aspect to be determined in a quantitative analysis for measuring the energy performance of a building envelope.

Recent IRT papers describing specific studies (not reviews) include the work by Barreira et al. [27], where the partial humidification of a lightweight concrete specimen is quantitatively evaluated through a thermal image sequence and by the application of a pre-processing step for data reduction, followed by a data processing step consisting of the application of different statistical and numerical methods. Furthermore, the automatic detection and delimitation of moisture areas affecting the surface is performed by analysing the temperature distribution of the thermal images acquired from several internal white plaster walls and on a concrete building façade [28]. With the same purpose of detecting moisture, the analysis of IRT data from a façade with adhered ceramic claddings is improved in another recent IRT work [29] using a thermal image processing algorithm known as Principal Component Thermography (PCT). Moreover, Lucchi et al. [30] and Andreotti et al. [31] present results referred to the hygrothermal monitoring of historic masonries. In both works, Temperature–Relative Humidity (RH) combined sensors and thermocouples are used together with IRT. The conclusions reached from these two works suggest the use of IRT for moisture control in addition to destructive tests.

With respect to GPR, Agliata et al. [32] present calibration equations linking the dielectric permittivity and the electrical conductivity to the water content of a full-scale wall made of Neapolitan yellow tuff bricks, demonstrating that both parameters are potentially good proxies to determine the water content of tuff bricks. In addition, Koyan et al. [33] estimate moisture changes in a reinforced concrete specimen using a diffraction-based velocity analysis. According to [34], building materials containing elevated amounts of moisture or moisture accumulation produce high-amplitude GPR signal reflections. Leucci et al. [35] include GPR measurements in order to investigate the moisture content inside a Crypt built in the middle of the 12th century with the capitals of the columns and walls built with a very porous Miocene limestone called *pietra leccese*. It was observed that water provokes the following effects on the GPR signal: (i) its high dielectric permittivity causes a decrease in the propagation velocity, and (ii) the high moisture content causes the attenuation of the EM energy, determining an increase in the electric conductivity. Consequently, the penetration depth can be highly reduced, and few or no reflections are observed beyond moisture zones. Interesting results are also found when mapping water leaks in the subsurface with GPR [36], showing that water distorted the signal due to an increase of the medium permittivity below the position of the leak in two different forms: (i) a reduction of the reflection amplitude beneath the leak position because of the attenuation of the electromagnetic waves traveling through the wetter area and (ii) a delay in the reflection time due to a decrease of the wave velocity. Other relevant studies regarding the analysis of the different amplitude values of the reflected pulses are found: (i) the effect of moisture on two different walls of an early 20th century building composed of both ancient adobe and modern bricks [37], (ii) the presence of a strong anomaly associated to the presence of a high amount of moisture in a specific part of an interior vault of a room from the early 19th century [38], (iii) the generation of moisture maps of the walls and floor of a residential basement made of lightweight aggregate blocks [39], and (iv) the evaluation of moisture levels in a thick travertine wall of an ancient church in Italy thanks to the “time-stretched signal”, “ringing” effects, and variations in the signal amplitudes [40].

There are also recent studies that combine IRT and GPR in building inspection. For example, Matera et al. [41] integrate GPR measurements with an IRT investigation performed on part of the vaulted ceiling of a church. The IRT dismisses the existence of intra-wall pipes or wires that were detected through the interpretations of some of the GPR results, consequently acting as a corrector. Lai and Poon [42] introduce IRT and GPR to facilitate building inspections, public housing blocks, and government buildings under the Mandatory Building Inspection Scheme (MBIS). The recognition of an anomalous signal from the GPR can be associated to the variation of material properties, such as wet or dry concrete. At the same time, the computation of the thermal gradient in the thermal images provides an estimate of the boundary and the size of the debond over the external wall envelope of high-rise buildings. Using different NDT techniques in addition to IRT and GPR, Martínez-Garrido et al. [43] perform a complete study to control moisture in a church based on the IRT rough estimates of the extent of moisture on the wall surfaces and the qualitative information on the areas where in-depth studies using GPR are performed. Moropoulou et al. [44] corroborate the results of IRT and GPR while analysing a monastery, as for example in the discontinuity at the interface of two walls. Nuzzo et al. [45] present the results on the rose window of a cathedral affected by widespread decay and instability problems caused by an earthquake. The determination of the internal structure of the circular ashlar curb and the presence of cracks in the columns and calcarenite elements with intersecting arches, as well as of the boundaries between original and restored parts, is possible with the GPR survey. Conversely, the detection of more superficial defects (cracks and superficial metal elements) and past conservation treatments (mortar-filled voids) is made through the IRT survey. Finally, Piroddi et al. [46] describe the preliminary results of integrated non-destructive surveys for the diagnosis of the materials and for the analysis of the underground structures of a historical building, including IRT and GPR. A 3D reconstruction of the buried structures and several buried anomalies localised under the floor level of two rooms of the building is obtained with the GPR, while discontinuities and more superficial defects on the investigated surfaces are found with IRT.

### 1.2. Benefits of IRT and GPR Applied to Building Inspection

As concluded from the analysis of the literature presented in the previous subsection, the combined application of IRT and GPR allows complementing the benefits of both techniques, as shown in Table 1.

### 1.3. Motivation

Although there are several published papers focusing on the combination of IRT and GPR as previously discussed, the particular application of IRT-GPR as complementary techniques has not been conveniently addressed on the current literature. In this framework, this work presents a new procedure to generate complementary information about the composition of the entire wall (integral surveying). In this context, the work is a contribution to the researchers and professionals on the data acquisition field toward an accurate and efficient monitoring. For this case study in particular, the combined approach herein enables estimating both superficial and internal moisture in buildings (also establishing a level of moisture severity), as well as predicting the water movement between the outside and the inside (or vice versa) and the relative depth of water accumulation or ingress. All this information, which is not reachable with only one technique, is meaningful data when designing prevention and maintenance tasks either for heritage or new buildings. Additionally, this methodological approach will be valid for in situ punctual or monitoring tests, as well as for laboratory tests. 

**Figure 1:**
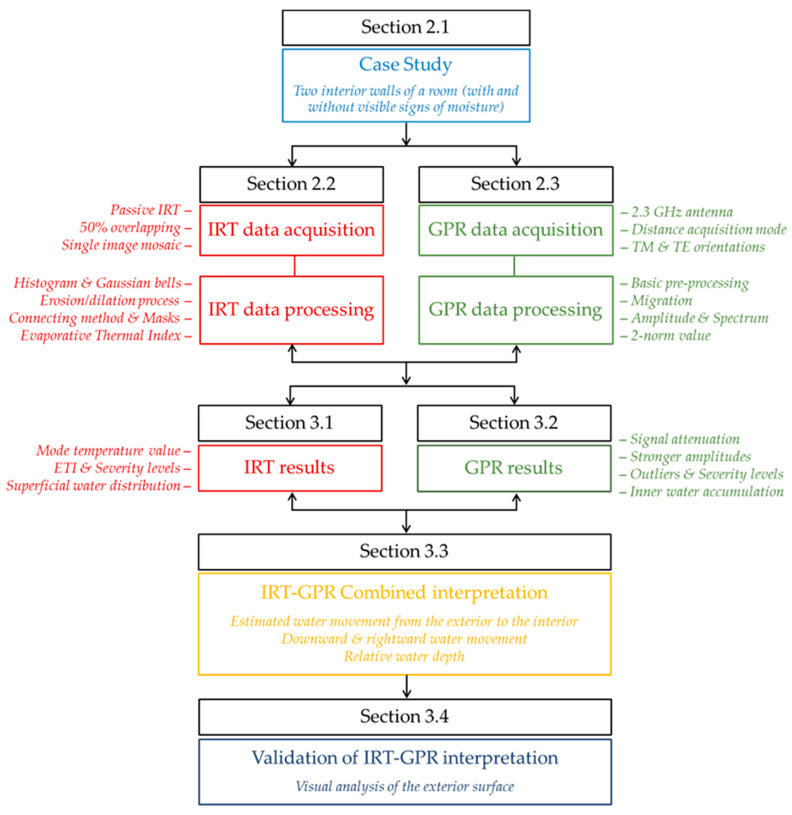
Structure and methodological workflow proposed.

In this particular case, an in situ punctual test is addressed, in which the proposed methodology is applied on two interior walls of a building constructed during the first half of the last century that shows visible signs of moisture. Figure 1 describes the structure and the methodological workflow followed in this work. 

## 2. Materials and Methods

### 2.1. Case Study

**Figure 2:**
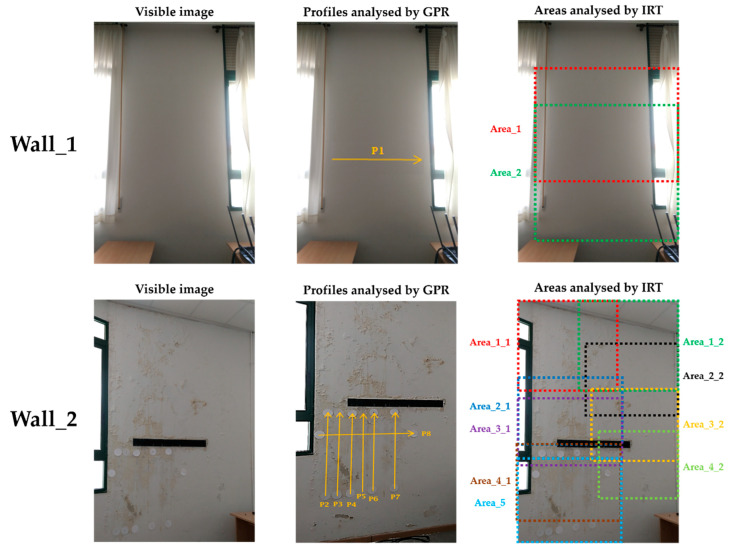
Visible images, areas analysed with IRT and profiles performed with GPR on the walls studied (1:75 scaling for wall).

The case study consists of two walls belonging to the envelope of a building constructed during the first half of the 20th century and located in a coastal zone. The walls are placed in an interior courtyard, away from the direct influence of the salty environment. They are contiguous walls belonging to the interior of classroom, but the study was performed during summer holidays, and the room had been empty for a month. The study focuses on the interior surfaces of the walls, as their state determines the level of indoor comfort and the possibility of health problems for the users. Figure 2 shows a visible image of the two walls studied, together with the indication of the areas analysed by IRT (indicated as ‘Area’ in the figure) and profile lines analysed by GPR (represented with ‘P’ in the figure). The areas and profiles were determined according to the degree of visible deterioration, dividing the surface in a higher number of areas and profiles on the most damaged zones with respect to zones with a low level of damage or apparently healthy.

An initial visual analysis shows no moisture affecting the surface of one of the walls under evaluation (Wall_1 in Figure 2), which will be assessed as a hypothetically healthy wall. However, deterioration is easily visible on the surface of the other wall studied (Wall_2 in Figure 2), showing a certain detachment of the plaster layer.

Then, all the zones on Wall_2 visibly affected by moisture are evaluated with IRT-GPR in order to confirm the diagnosis, as well as to define the extent and depth of the moisture areas. An estimation of moisture severity was also provided for each of the moisture areas detected. The GPR profiles are only centred on the region below the rack (the most visibly deteriorated zone of the Wall_2) because of the technical difficulties related to accessing the upper parts of the Wall_2 caused by the excessive height, the weight of the antennas, and the need to maintain the GPR antenna in contact with the surface.

It is also important to mention that both IRT and GPR data acquisitions were performed at noon, with outside/inside ambient temperature values and outside/inside ambient RH values equal to 22 °C/21.8 °C and 69%/50%, respectively. 

### 2.2. IRT Data Acquisition and Processing

**Figure 3:**
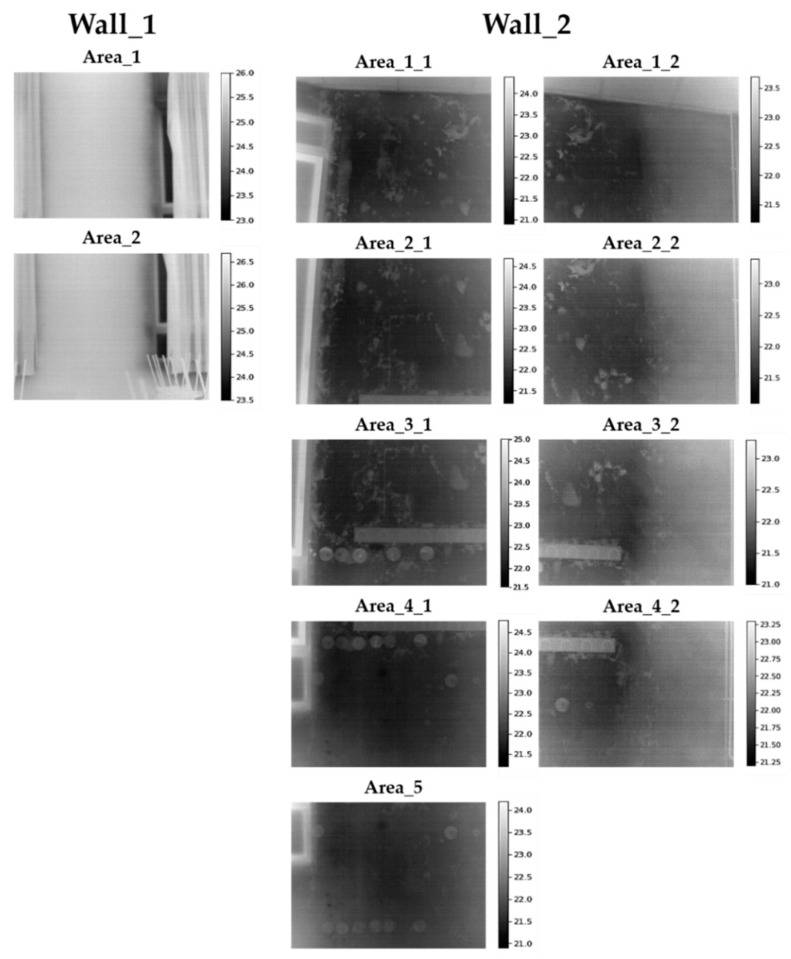
Thermal images acquired from Wall_1 and Wall_2 (corresponding temperature scales in °C at the right). 1:75 scaling for each thermal image.

Figure 3 shows the thermal images acquired on the areas analysed of both Wall_1 and Wall_2 indicated in Figure 2 (with the corresponding temperature scales in °C at the right).

The specifications of the IR camera used for the acquisition of the thermal images are detailed in Table 2.

Accurate temperature values are needed to define the different levels of moisture severity. Therefore, the correction factors for emissivity, reflected temperature, and atmospheric attenuation are applied to all the thermal images. The emissivity value of the plaster surface and the reflected temperature value are set to 0.93 [58] and 21.8 °C, respectively, and the atmospheric attenuation is set to 0.01, taking into account that the distance between the IR camera and the areas analysed is around 1.5 m and that the inside ambient temperature/RH values are 21.8 °C and 50%, respectively (as seen in Section 2.1). 

Observing Figure 3 in detail, the thermal images acquired have been overlapped by about 50% for their integration into a single image mosaic representing the whole wall. In this way, a complete visualisation of each thermally interesting area and of the thermal condition of Wall_1 and Wall_2 is achieved, avoiding missing defects that appear partially on the different thermal images. In other words, the overlapping zones allow the identification of common features among the thermal images. For the overlapping, the thermal images have been acquired placing the IR camera as perpendicular as possible to the surfaces under study, and reflective targets have been placed to facilitate the identification of common points among the thermal images, as well as the association between the thermal images and the GPR profile lines (see Figure 2 and Figure 3). Reflective sheets were used as reflective targets, which were placed only on Wall_2 because of the high number of areas analysed with IRT. In the case of Wall_1, its division in only two areas allows for the determination of the overlapping area without using reflective targets.

The processing steps described in the following subsections are performed for each thermal image using Python 3.5 as a programming language. It should be noted that all the steps are based on the fact that areas affected by moisture produce a Gaussian temperature distribution on the histogram of the thermal images, which is independent of the Gaussian temperature distribution presented by the unaltered zones. This assumption has been proved in previous papers published by the authors of this work [20,28,49,59]. In addition, the thermal images are automatically processed in all the steps.

#### 2.2.1. Step 1: Determination of Ends of Possible Gaussian Bells

**Figure 4:**
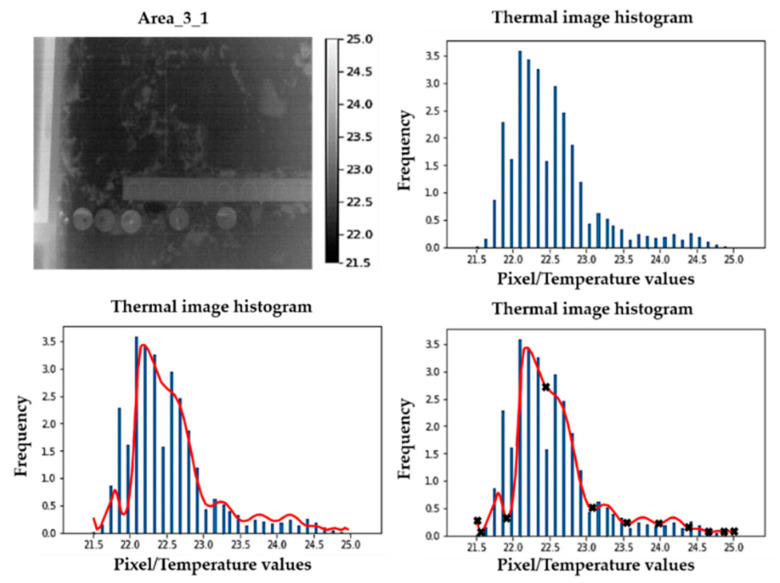
First row: thermal image of Area_3_1 of Wall_2 and its corresponding histogram. Second row: Savitzky–Golay (SG) application on the previous histogram (red line) and the corresponding relative minimum values, inflection points, and extremes of SG output identification (black crosses).

The first step determines the extremes of possible Gaussian bells in the histogram of the thermal image. To do this, the Savitzky–Golay (SG) smoothing filter [60] is applied. This filter tends to preserve characteristics of the initial temperature distribution of the thermal image (i.e., its histogram) such as relative maximum/minimum values and inflection points while softening the intermediate values. The output of the SG filter is a line that fits the shape of the thermal image histogram discarding the noise and preserving the ends of possible Gaussian bells, that is, relative minimum values and inflection points. Then, the relative minimum values and the inflection points are identified, highlighting that the ends of the SG output line are also considered as ends of possible Gaussian bells. As an example, Figure 4 shows the result of Step 1 applied to the thermal image acquired from Area_3_1 of Wall_2.

#### 2.2.2. Step 2: Erosion/Dilation Process and Connecting Method Application

With the identification of the ends of possible Gaussian bells on the thermal image histogram, a mask image is generated for each interval between two consecutive ends. As a result, the pixel values of the image outside/inside the corresponding interval are equalised to zero/one, respectively. Therefore, several mask images are generated, with two groups of pixels: (i) those equal to zero (represented in black), (ii) and those equal to one (represented in white). Regions in white (pixels equal to one) are considered to be candidates for superficial moisture areas and then are subjected to an erosion/dilation process and to a connecting method. Regions in black (pixels equal to zero) are considered as no-moisture and consequently dismissed from any further processing. With the erosion process, very small regions considered as noise are eliminated, while the dilation process groups the closest regions into a single region. After the erosion/dilation process, each region is labelled with a different index in the connecting method. More explanation about the erosion/dilation process and the connecting method can be found in [61] and [62], respectively.

**Figure 5:**
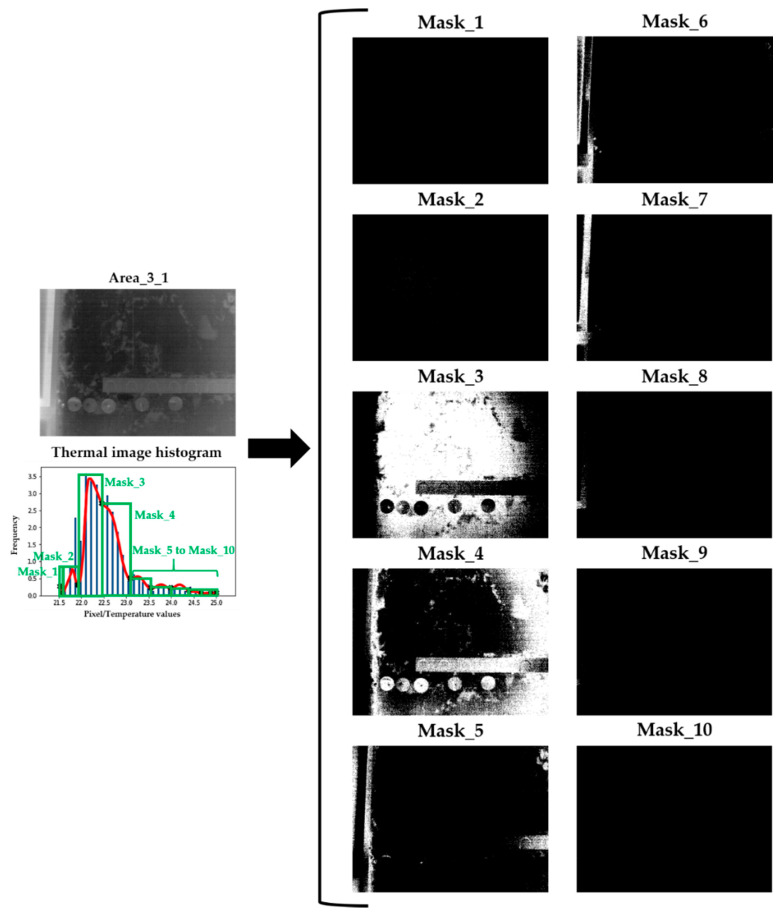
Zones of the thermal image of Area_3_1 of Wall_2 corresponding to each of the possible Gaussian bells (mask images). Each mask image contains one or more regions (white areas in the figure) that are considered as candidates for superficial moisture areas.

**Figure 6:**
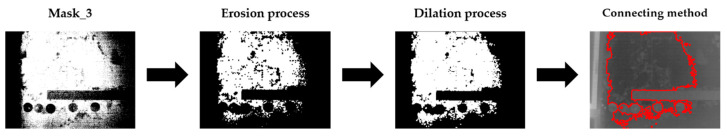
Erosion/dilation process and connecting method applied to Mask_3 of Figure 5. The contours of the different candidates for superficial moisture areas are delimited by a red line after the application of the connecting method.

Figure 5 shows the mask images generated according to the identifications of the ends of possible Gaussian bells represented in Figure 4. In turn, Figure 6 shows the result of the erosion/dilation process and the connecting method applied on one of the mask images in Figure 5 (Mask_3).

#### 2.2.3. Step 3: Removing of Candidates for Superficial Moisture Areas with False Gaussian Bells

According to [28], the distribution of a dataset can be considered Gaussian as long as it has values of skew and kurtosis between −2 and +2. The skew parameter consists of the degree of distortion of the distribution of a dataset regarding a symmetrical distribution, i.e., the farness or closeness to the zero skew value, which is the corresponding value for an ideal Gaussian bell. On the other hand, the kurtosis parameter analyses the tails in a data distribution with respect to the tails of a Gaussian bell, being zero kurtosis value if they have the same tails [49].

Therefore, the skew and kurtosis values of the candidates to be superficial moisture areas obtained after the application of the Step 2 are calculated. In this way, regions with false Gaussian temperature distributions, i.e., with skew and/or kurtosis values outside the range −2 to +2, are considered as false candidates for superficial moisture areas (i.e., considered as noise or some artefact) and consequently discarded from further processing. 

**Figure 7:**
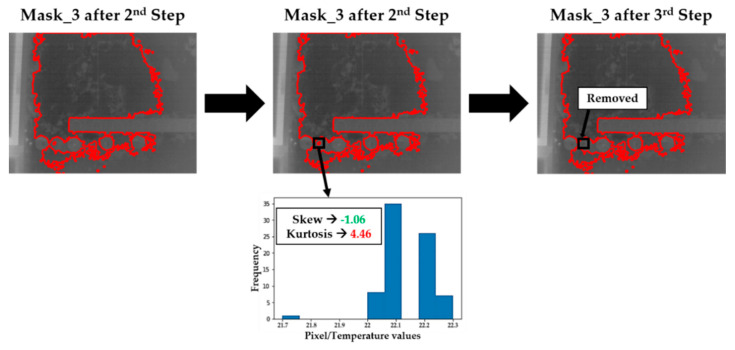
Removal of candidates to be superficial moisture areas of the mask image represented in Figure 6 with false Gaussian temperature distributions.

**Figure 8:**
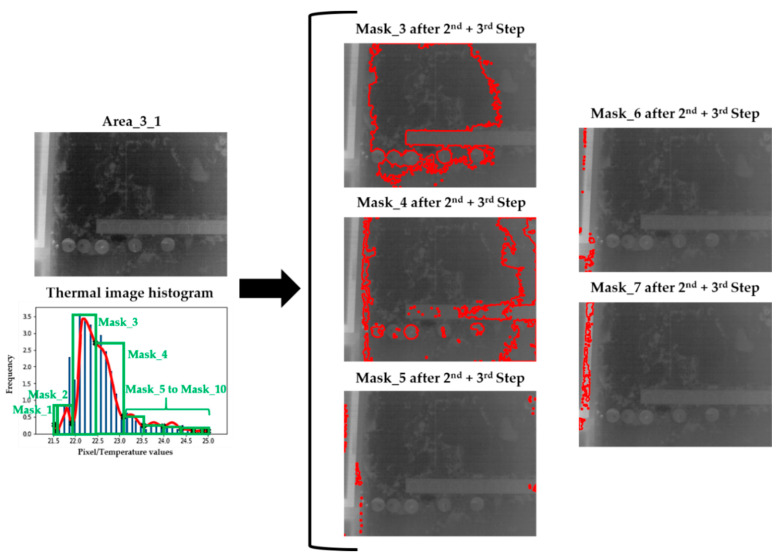
Result of the application of the Steps 2 and 3 on the zones of the thermal image of Area_3_1 of Wall_2 corresponding to each possible Gaussian bell. The candidates for superficial moisture areas are represented by the delimitation of their contours (red lines). The mask images that do not appear in this figure do not present any moisture candidates after these steps of the methodology.

Figure 7 illustrates the performance of Step 3 on the dismissal of moisture-candidate zones in Mask_3 of Figure 6, whereas Figure 8 shows the result of the application of the Steps 2 and 3 on the zones of the thermal image of Area_3_1 of Wall_2 corresponding to each of the possible Gaussian bells.

#### 2.2.4. Step 4: Removal of Candidates for Superficial Moisture Areas According to Their Mode Temperatures and the Ambient Temperature

As explained in Section 2.2, the unaltered zones can also present a Gaussian temperature distribution, as can be seen in some regions delimited in Figure 8 which correspond to the (i) reflective targets, (ii) rack, (iii) wall, (iii) window frame, and (iv) window glass. Moreover, it is considered that both Wall_1 and Wall_2 are in a quasi-stationary state due to the absence of use of the room from one month prior to the acquisition, which is proved by the fact that the inside (21.8 °C) and the outside (22 °C) of the building present practically the same temperature value. There is a slight increase of the outside ambient temperature from sunrise to the instant of the test campaign (the acquisitions were made at noon), at which point the temperature difference (from 15 to 22 °C) is considered negligible in comparison to the temperature difference considered for active IRT [54]. 

In this way, an evaporation process occurs on the moisture areas in the case of the presence of water on the Wall_1 and Wall_2. As the evaporation process is an endothermic reaction that induces a decrease in the temperature of the area affected by the moisture with respect to its unaltered surroundings [28], the temperature of the superficial moisture areas is considered to be lower than the inside ambient temperature. Instead, the temperature of the unaltered zones of Wall_1 and Wall_2 is considered to be higher than the inside ambient temperature, due to the heat accumulation in the material. 

**Figure 9:**
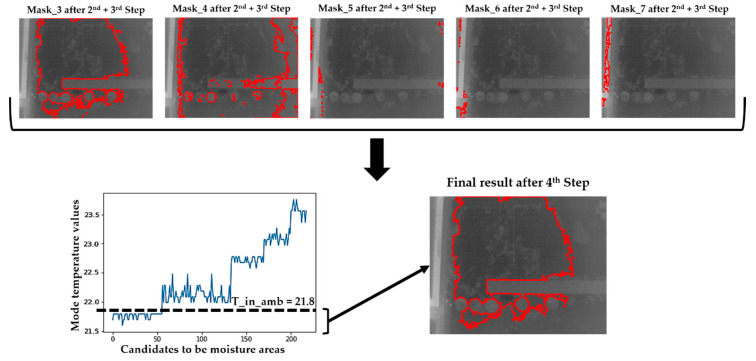
Result after the application of the Step 4 on Figure 8, representing the final candidates for superficial moisture areas by the delimitation of their contours (red lines). ‘T_in_amb’ means inside ambient temperature.

It should be noted that the inside ambient temperature is used as the threshold value for the two previous considerations, because in coastal climate conditions, the thermal difference between the outside and inside of a building is low. This implies that the temperatures of the superficial moisture areas and of the unaltered zones are slightly lower and higher than the inside ambient temperature during a heating phase, respectively (i.e., from sunrise until noon). Specifically, the difference in apparent temperature is usually up to a few degrees Celsius between the inside ambient temperature and the temperature of unaltered zones (maximum 2–4°C) because of the fact that some unaltered zones may have surfaces with low emissivity values, thus reflecting the heat incident to the walls in the thermal images (e.g., artificial lighting). However, the temperature difference between the inside ambient temperature and the temperature of the superficial moisture areas is usually a few decimals of degrees Celsius, as the emissivity of the water is close to 1 [63] and with the temperature difference value directly proportional to the water accumulated in the moisture area. So, candidates that have a temperature value at the maximum peak of their Gaussian temperature distributions higher than the inside ambient temperature value are considered as unaltered zones and, consequently, discarded as final candidates. In other words, candidates with a mode temperature value (i.e., most frequent temperature value) lower than the inside ambient temperature value are considered as true superficial moisture areas. Figure 9 shows the final candidates grouping all the mask images into one for the thermal image of Area_3_1 of Wall_2. Figure 9 shows the magnitude of the following temperature differences: (i) between the inside ambient temperature and the temperatures of the unaltered zones, and (ii) between the inside ambient temperature and the temperatures of the superficial moisture areas. The highest temperature difference appears with the window frame, which is made of aluminium: a material with low emissivity [64].

#### 2.2.5. Step 5: Moisture Severity Levels: Evaporative Thermal Index (ETI) Calculation on Each Candidate for Superficial Moisture Area 

After obtaining the final candidates, Step 5 consists of their characterisation. Specifically, the parameter Evaporative Thermal Index (ETI) is calculated for each final candidate in order to establish different levels of moisture severity. The ETI was proposed by Tavukçuoğlu and Grinzato [65] and Grinzato et al. [66], and it is defined as the estimation of the evaporation rate on the superficial moisture area, allowing the calculation of the critical moisture content of materials without considering the distribution of moisture inside the surface [27]. Therefore, the ETI parameter allows the identification of the most severe areas of superficial moisture and the establishment of different levels of deterioration on the zones affected. The ETI is obtained using the following equation:

ETI = |*T_moisture_area_* − *T_dry_surface_*|/*T_dry_surface_* (1)

where *T_moisture_area_* and *T_dry_surface_* are the temperature value of the superficial moisture area and dry surface/unaltered surrounding in the same units, respectively. In this case, the mode temperature value of a final candidate is input as *T_moisture_area_*, and the inside ambient temperature is input as *T_dry_surface_* due to the small difference between the outside/inside ambient temperature.

**Figure 10:**
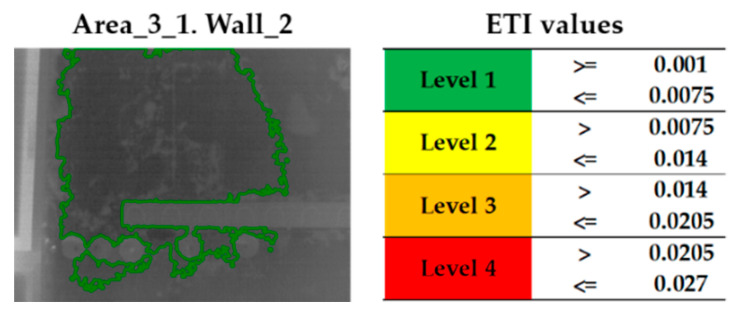
Evaporative Thermal Index (ETI) calculation for each final candidate to be a superficial moisture area on the thermal image of Area_3_1 of Wall_2 and colour labelling according to the moisture severity level.

Figure 10 shows the result obtained after the application of this Step 5 on the thermal image of Area_3_1 of Wall_2. Figure 10 (left) shows the contours of the final candidates, which are delimited by applying a specific colour according to their ETI values; Figure 10 (right) includes the legend of each colour applied to the contours indicating which range of ETI values is covered. The different ranges of ETI values are specifically designed for the case study, consisting of the equal division of the range between the minimum ETI value and the maximum ETI value of all the thermal images analysed in four levels. In this way, a colour is assigned to a specific moisture severity level, in such a way that the colours act as a traffic light: (i) red colour indicates the superficial moisture areas with the highest ETI level (highest moisture severity level, “Level 4”), (ii) orange and yellow indicate the superficial moisture areas with intermediate ETI levels (intermediate moisture severity levels, “Level 3” and “Level 2”) and (iii) green indicates the superficial moisture areas with the lowest ETI level (lowest moisture severity level, “Level 1”).

The final candidates with the corresponding ETI results and colour labelling according to the moisture severity level from the other thermal images acquired in this work are represented and discussed in Section 3.1 together with the result shown in this section.

### 2.3. GPR Data Acquisition and Processing

The GPR method was employed to assist in the detection of moisture in the interior of the walls, which is helpful for the investigation of damage in building materials or pathologies in construction. Seven GPR profiles were acquired, which are spatially distributed on Wall_2 (Figure 2). Additionally, one GPR profile was gathered along Wall_1 (Figure 2) in order to confirm whether the wall is affected by moisture or not. If not affected, this profile will be used as ground truth or reference to compare with the GPR data produced for Wall_2.

**Figure 11:**
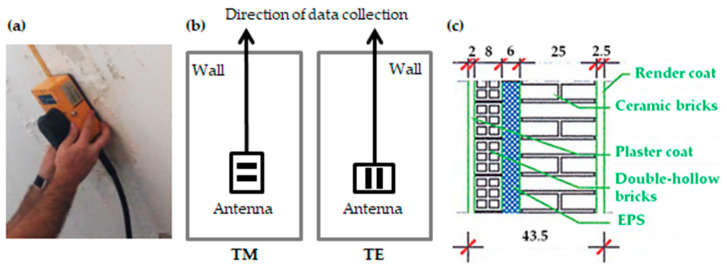
GPR data acquisition: (**a**) 2300 MHz GPR antenna and tape measure used for a point-by-point data acquisition, (**b**) Transverse Magnetic (TM): data acquisition with the antenna in the normal survey configuration (perpendicular broadside orientation with the transmitting and receiving dipoles perpendicular to data collection direction) and Transverse Electric (TE): data acquisition with the antenna rotated 90° (parallel broadside orientation with the dipoles parallel to data collection direction), and (**c**) wall composition (sizes in cm).

A ground-coupled pulsed system manufactured by Malå Geoscience© was used, which was composed of a Proex control unit and bistatic antennas, having a central frequency of 2300 MHz. The setup used for data acquisition was a trace-interval of 0.01 m and time window of 14 ns composed of 292 samples per trace. To measure the profile lengths, and to control the distance between traces, a tape measure was used as a guide (Figure 11a).

All the GPR profiles were produced in two different acquisition modes: (i) with the Transverse Magnetic (TM) polarisation (Figure 11b-TM), and (ii) with the Transverse Electric (TE) polarisation (Figure 11b-TE). Even though the normal survey acquisition mode is the TM orientation, there are previous studies that have reported better horizontal resolution in the nearest subsurface with the TE orientation [67,68]. Therefore, both dipole orientations were herein tested.

Before interpretation, all the GPR data were processed with ReflexW software© (Sandmeier geophysical research, Karlsruhe, Germany) [69] using the processing sequence described in Table 3. This filtering aimed to eliminate possible noise or interference with the signal, as well as to amplify the received signal (Gain Function) in order to mitigate possible losses or attenuations. To suppress the continuous component, a vertical or temporal filtering was applied (Subtract-mean-Dewow), which allowed obtaining and eliminating from each trace an average value based on the low energy of the trace coda. A horizontal or spatial filtering (Subtracting average) was also applied to remove horizontal continuous low-frequency reflectors, which allowed estimating and removing an average value of all the traces in a time window. Finally, migration processing (Kirchhoff) was used to suppress strong clutter.

**Figure 12:**
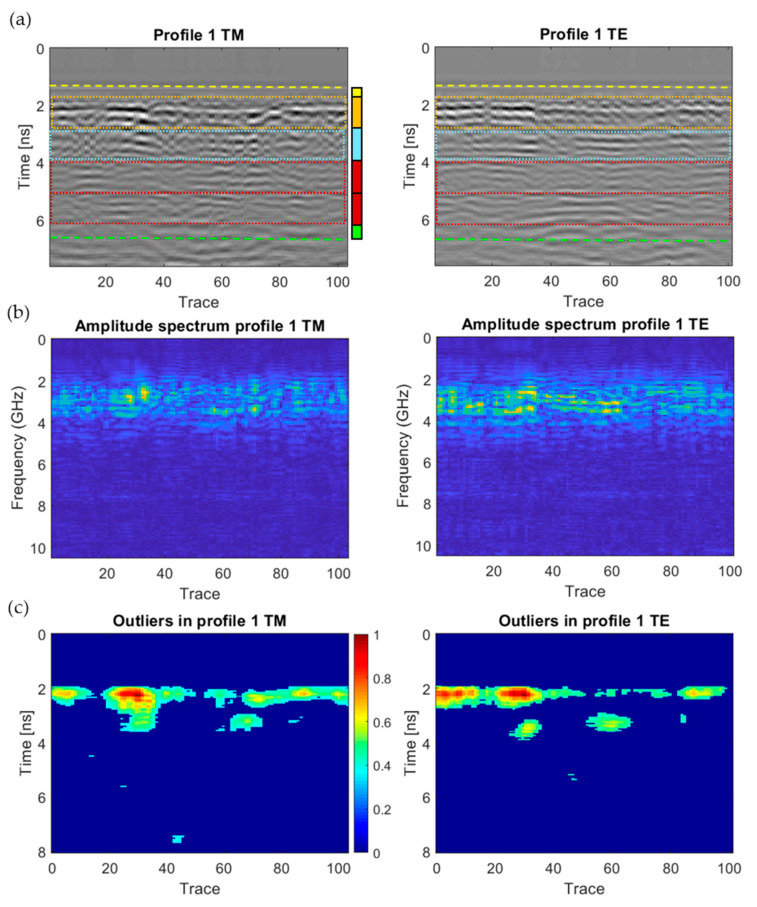
Graphics obtained for GPR profile 1 (Wall_1): (**a**) radargram (amplitudes in greyscale) showing the interpretation of building materials: plaster coat (yellow rectangle), one layer of double hollow bricks (orange rectangle), Expanded Polystyrene (EPS) boards (blue rectangle), double layer of ceramic bricks (garnet rectangles), and render coat (green rectangle), (**b**) amplitude spectrum of the radargram (colour-scale map), and (**c**) normalised amplitude value of the outliers (same scale for both subplots).

In order to assist with the analysis and interpretation of the GPR signals received, different algorithms were developed based on signal attributes such as amplitude and its spectrum. As an example to describe the algorithms elaborated, Figure 12 presents the GPR data obtained for Profile 1, which is the one recorded through the wall without visible external signals of moisture (Wall_1 from Figure 2). First, Figure 12a represents a conventional radargram (XZ image) where the amplitude of the received echo is shown in greyscale, the trace number is on the X axis, and the travel time of the radar signal (in nanoseconds) is on the Z axis. Although the total time window is near 14 ns, only the first 160 samples of each trace (around 8 ns) are represented. The aim is to focus on the area where all the relevant features are placed. Next, Figure 12b shows a colour-scale XZ map of the one-sided amplitude spectrum of the radargram, with the trace number on the X axis and the frequency (in GHz) on the Z axis. This figure is obtained by applying the Fast Fourier Transform (FFT) function from Matlab^®^ to the time-domain signal of each trace and representing the absolute value of the positive side of the spectrum. Note that the continuous component (frequency 0) has been removed, by subtracting the mean from the time-domain signal of each trace before computing its FFT. Finally, Figure 12c shows the outliers detected in the radargram of the Profile 1 for both the TM (left) and TE (right) modes. This figure is obtained as follows:

1 The radargrams of the TM and TE modes are filtered with an averaging 2D filter working on a 7 × 7 window.
2 Then, the absolute value of the matrix obtained from the filtering process is computed.
3 This matrix is later normalised in the [0,1] interval dividing all the cells by the maximum value of the matrix.
4 Finally, the outliers in the normalised matrix are detected using the InterQuartile Range (IQR) criterion: those values that are greater than the upper 1.5 times IQR whisker or lesser than the lower 1.5 times IQR whisker are considered outliers and plotted, using a colour-scale shown on the right side of the TM subplot.

Observing all the GPR products obtained for Profile 1, in Wall_1 (visibly non-damage area), both the yellow and green dashed lines in Figure 12a, at ≈1.5 ns and 6.5 ns, respectively, enclose the wall thickness (43.5 cm in Figure 11c), which is composed of (from interior to exterior): plaster coat (2 cm, ≈0.5 ns thick), one-layer of double hollow bricks (8 cm, ≈1 ns thick), two Expanded Polystyrene (EPS) insulation boards (6 cm, ≈1 ns thick), a double layer of ceramic bricks (25 cm, ≈2 ns thick) and render coat (2.5 cm, ≈0.5 ns thick). The reflections produced are continuous and in the form of layering through the entire radargram. Moreover, the reflection generated from the wall thickness appears stable at the same depth, which means that the dielectric constant is relatively homogenous through the media. Note that although the survey paths are vertical, except for Profile 8 (see Figure 2), the corresponding coordinates in the radargrams are horizontal. Regarding the amplitude spectrum of the radargram (Figure 12b), the higher values (yellow colours in graphs (b)) are obtained through the bricklayer (the one-layer of double hollow bricks), at ≈2.5 ns, which is more certainly due to the scattering of the signals produced by the shallow air gaps in bricks. A similar behaviour is observed in graph (c), in which the higher amplitude values of the outliers (identified as red colours) were produced at the bricklayer. 

Additionally, a boxplot was computed in order to compare the GPR signal behaviour at both the visible non-damaged area (Wall_1) and damaged area showing external signs of moisture (Wall_2). The boxplot represents the distribution obtained by computing the 2-norm of each of the vertical traces in the radargram of a profile.

**Figure 13:**
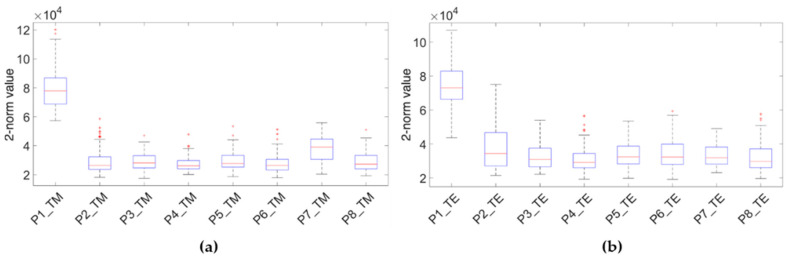
Boxplots comparing the profile (P1) within the visible non-damaged area (Wall_1) with the profiles (P2–P8) within the area showing external signs of moisture (Wall_2): TM (**a**) and TE (**b**) modes.

Figure 13 presents the results obtained in both the TM (a) and TE (b) orientations, in which it is possible to observe that all the boxes from the damaged area have a similar distribution and appear overlapped (with the boxes at a similar range in the Y axis), while the box from the visibly non-damaged area appears at a higher range. This fact may be because the radar wave penetrates deeper in Wall_1 where there is no attenuation of the signal by moisture and, therefore, more reflections are produced in the coda; hence, the value of the median of the 2-norm of the traces increases. All the data obtained seem to confirm no moisture affection in Wall_1.

## 3. Results and Discussion

### 3.1. IRT Imaging and Data Interpretation

**Figure 14:**
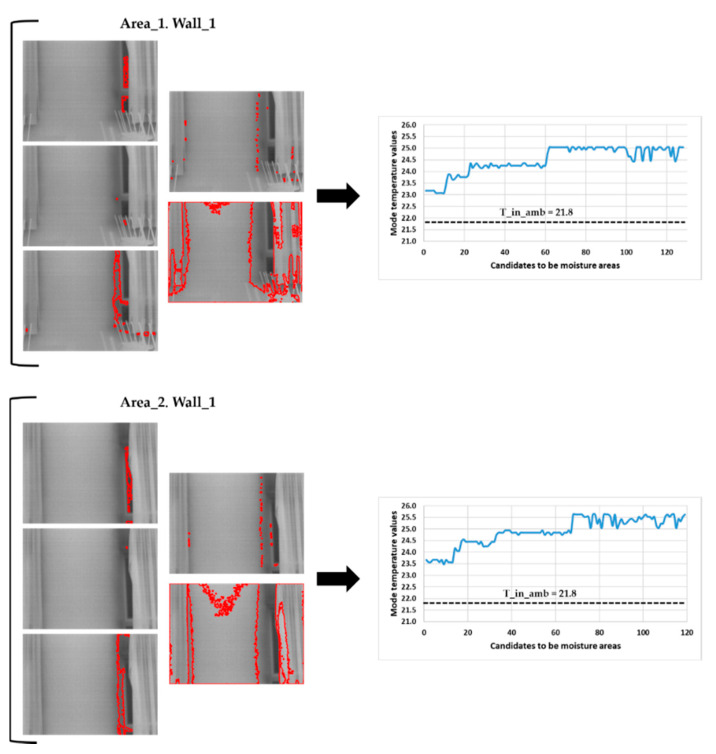
Result after the application of Step 4 of the IRT method on the thermal images of Area_1 and Area_2 of the Wall_1. ‘T_in_amb’ means inside ambient temperature.

Figure 14 shows the mode temperature value of each candidate to be a superficial moisture area after applying Step 4 of the IRT methodology on the thermal images of Area_1 and Area_2 of Wall_1.

Analysing the previous figure, all mode temperature values are higher than the inside ambient temperature value. This means that all the candidates on Area_1 and Area_2 of the Wall_1 are actually unaltered zones, confirming that Wall_1 is free of moisture, as shown in the visible image in Figure 2.

**Figure 15:**
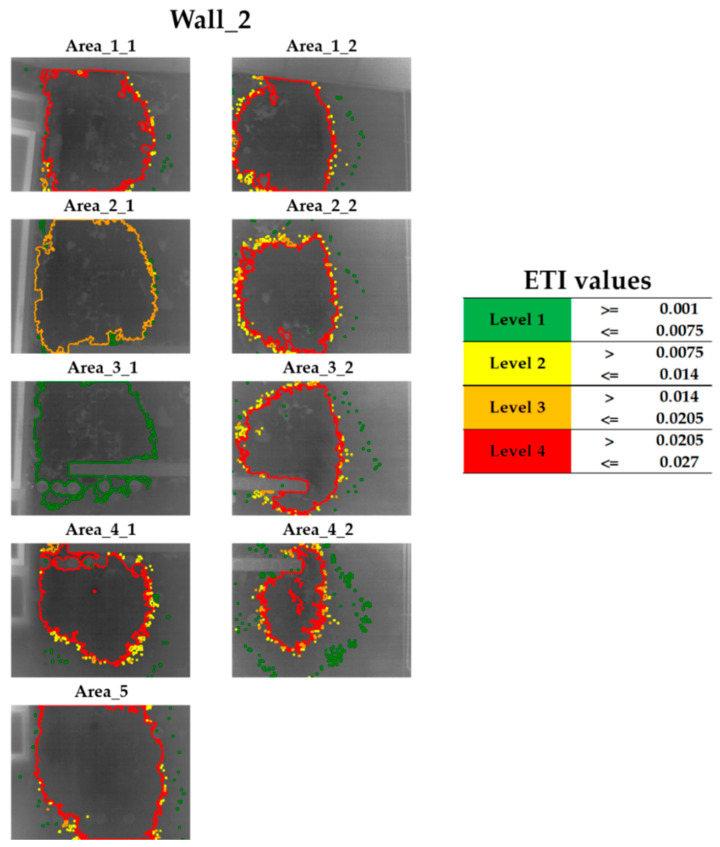
Final candidates for superficial moisture areas with the corresponding ETI values and colour labelling according to the moisture severity level obtained for each thermal image acquired on the Wall_2.

As for Wall_2, Figure 15 shows the final candidates for superficial moisture areas with the corresponding ETI results and colour labelling according to the moisture severity level.

**Figure 16:**
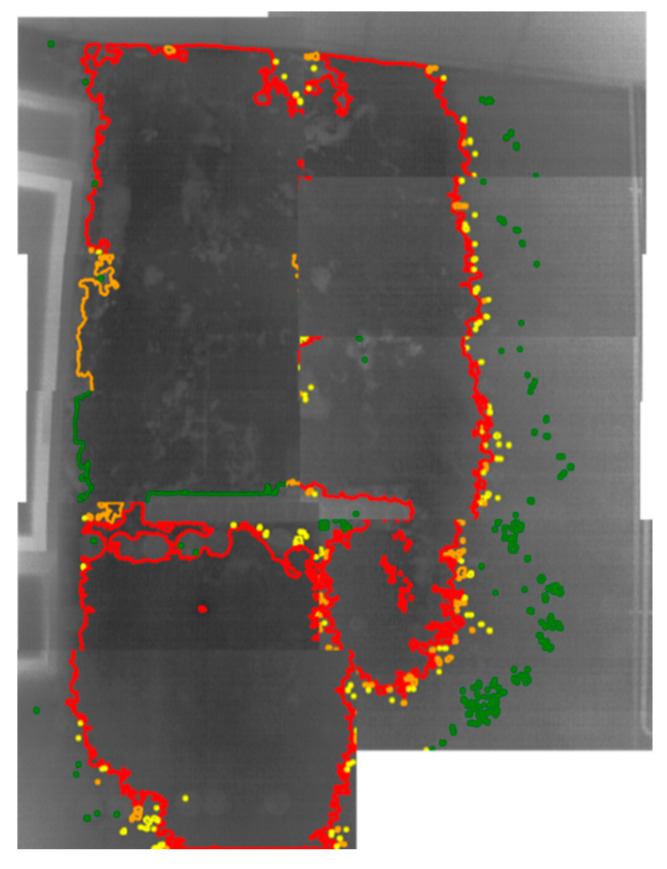
Merged representation of the thermal images in Figure 15.

It can be seen that a large superficial moisture area predominates in each of the thermal images. Specifically, the largest areas are located: (i) at the right side of the window frame, (ii) between the right side of the window frame and the rack, (iii) around the rack, and (iv) the zone from the top of the rack to the ceiling. Then, the top and bottom side of the window frame as well as the rightmost zone of Wall_2 are free of superficial moisture. Moreover, focussing on the overlapping zones of the thermal images (see the thermal images of Figure 15 merged into Figure 16), it is deducted that the predominant superficial moisture areas of the thermal images actually form one single area.

**Figure 17:**
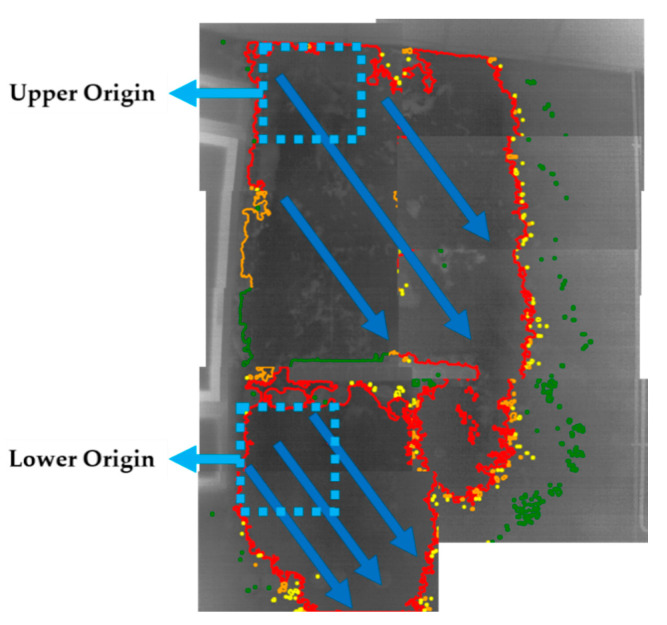
Superficial water distribution (blue arrows) through Wall_2 according to the shape and sections of the different colours of the contours in Figure 16.

Analysing the shape and the sections of the different colours of the contours in Figure 16, Figure 17 displays the more superficial distribution of water through Wall_2 (blue arrows).

The interpretation of the thermal results proposes a displacement of the water next to the surface with origin in two zones according to the path shown with the blue arrows, being each origin near to a different corner of the right side of the window frame (‘Upper Origin’ and ‘Lower Origin’ in Figure 17). From these origins, water goes downwards due to the effect of gravity as expanding to the right of Wall_2 but without reaching the rightmost zone. The justification for the rightward expansion without reaching the rightmost zone of Wall_2 is due to the lower degree of porosity in the shallow depths of the following zones despite having the same building materials along the entire wall: (i) above and below the window, (ii) the rightmost zone of Wall_2, and (iii) even between the right side of the window frame and the rack and in the central zone of the right side of the window frame due to their lower levels of moisture severity. The opposite occurs with the slight rise of superficial water to reach the ceiling from the ‘Upper Origin’ and to reach the bottom of the rack from the ‘Lower Origin’, although the bottom of the rack can also be affected by the water that starts from the ‘Upper Origin’ because it is in an upper position. Section 4 validates the IRT interpretation.

### 3.2. GPR Imaging and Data Interpretation

**Figure 18:**
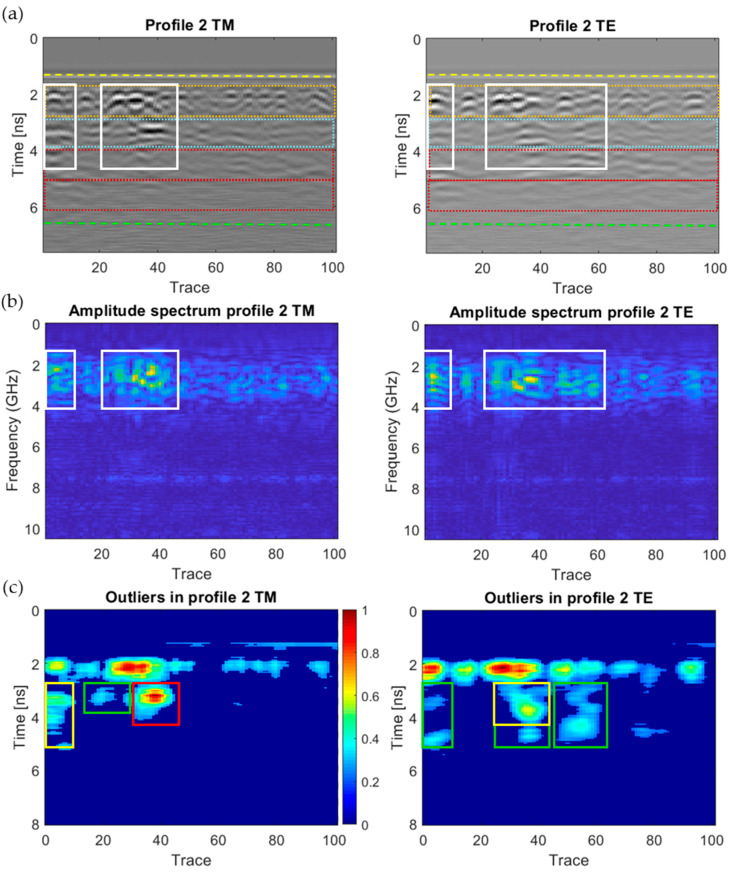
Graphics obtained for GPR profile 2 (Wall_2): (**a**) radargram (amplitudes in greyscale) (the colour code used in interpretation is the same as in Figure 12), (**b**) amplitude spectrum of the radargram (colour-scale map), and (**c**) normalised amplitude value of the outliers (the scale is the same for both subplots).

**Figure 19:**
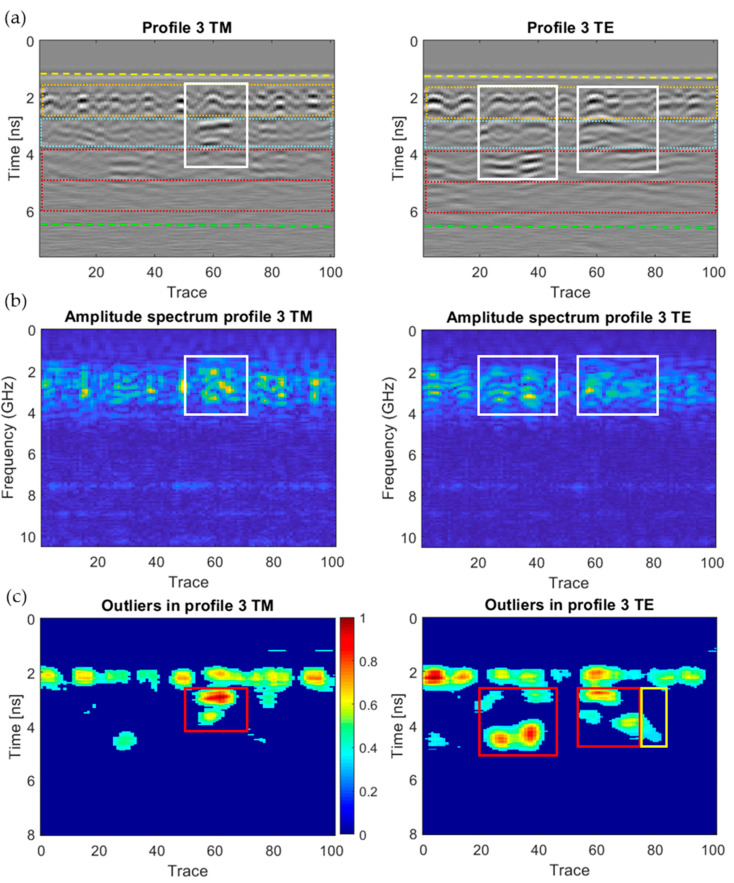
Graphics obtained for GPR profile 3 (Wall_2): (**a**) radargram (amplitudes in greyscale) (the colour code used in interpretation is the same as in Figure 12), (**b**) amplitude spectrum of the radargram (colour-scale map), and (**c**) normalised amplitude value of the outliers (same scale for both subplots).

**Figure 20:**
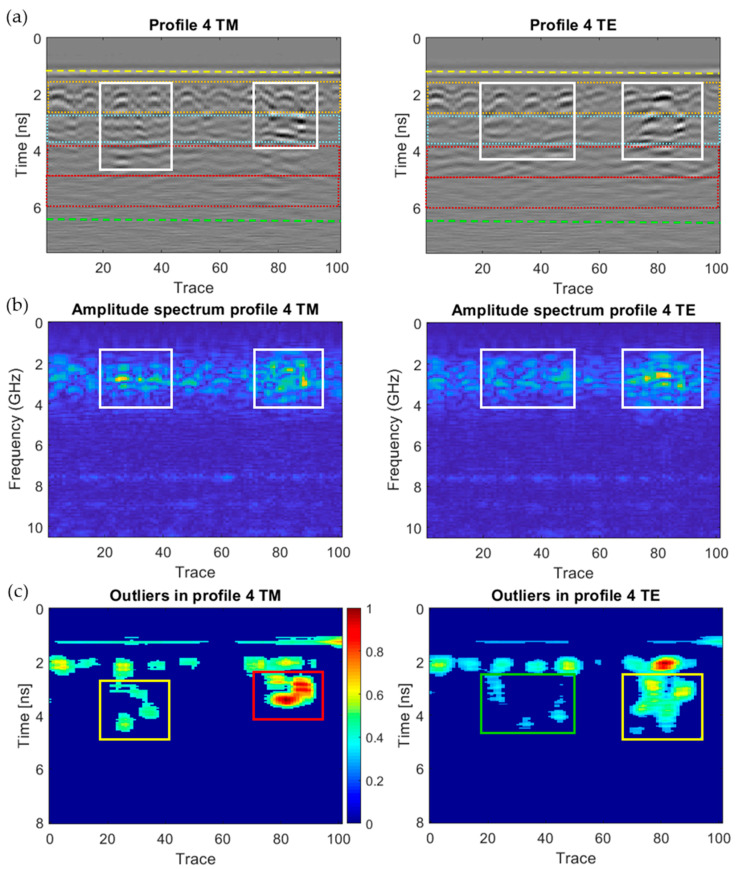
Graphics obtained for GPR profile 4 (Wall_2): (**a**) radargram (amplitudes in greyscale) (the colour code used in interpretation is the same as in Figure 12), (**b**) amplitude spectrum of the radargram (colour-scale map), and (**c**) normalised amplitude value of the outliers (same scale for both subplots).

**Figure 21:**
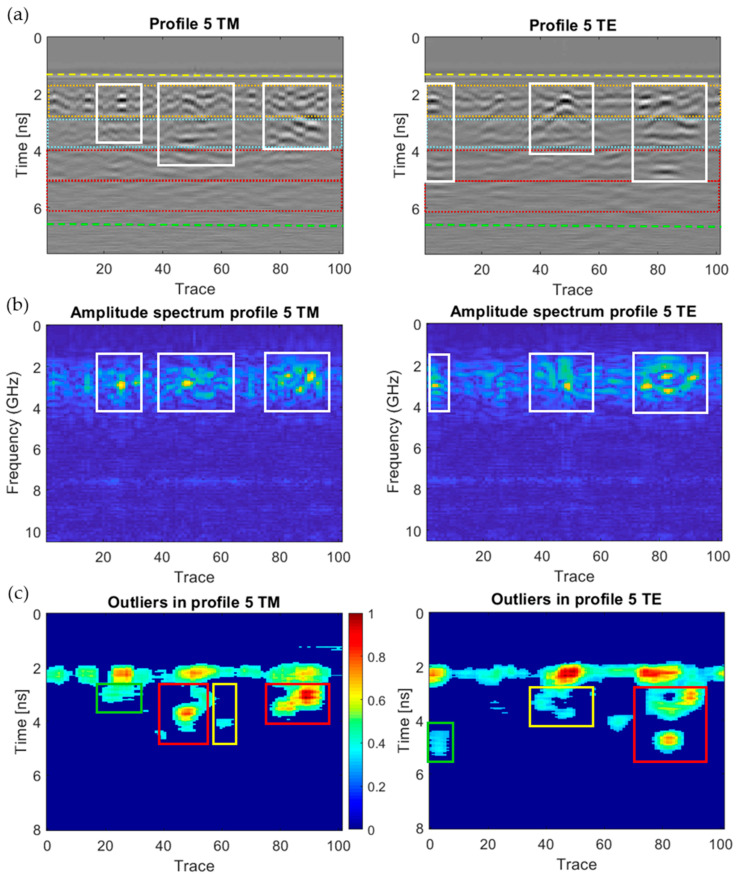
Graphics obtained for GPR profile 5 (Wall_2): (**a**) radargram (amplitudes in greyscale) (the colour code used in interpretation is the same as in Figure 12), (**b**) amplitude spectrum of the radargram (colour-scale map), and (**c**) normalised amplitude value of the outliers (same scale for both subplots).

**Figure 22:**
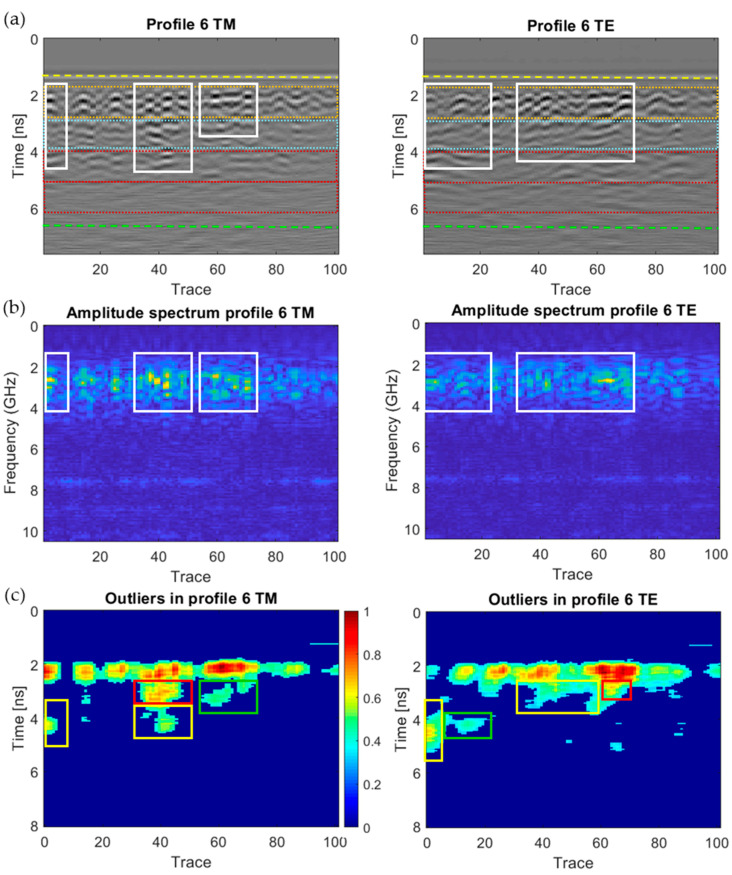
Graphics obtained for GPR profile 6 (Wall_2): (**a**) radargram (amplitudes in greyscale) (the colour code used in interpretation is the same as in Figure 12), (**b**) amplitude spectrum of the radargram (colour-scale map), and (**c**) normalised amplitude value of the outliers (same scale for both subplots).

**Figure 23:**
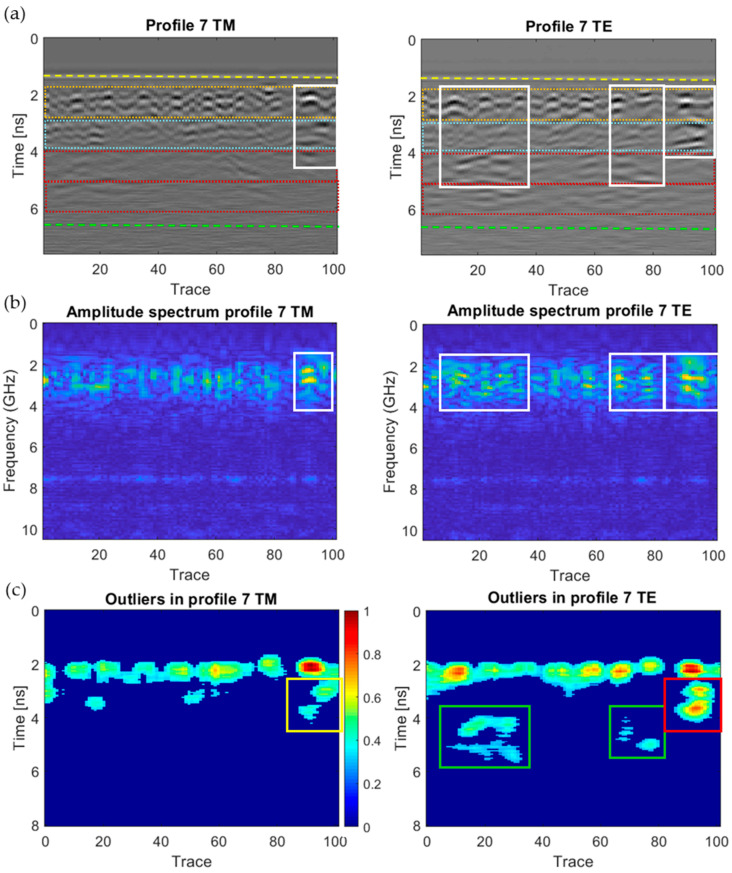
Graphics obtained for GPR profile 7 (Wall_2): (**a**) radargram (amplitudes in greyscale) (the colour code used in interpretation is the same as in Figure 12), (**b**) amplitude spectrum of the radargram (colour-scale map), and (**c**) normalised amplitude value of the outliers (same scale for both subplots).

**Figure 24:**
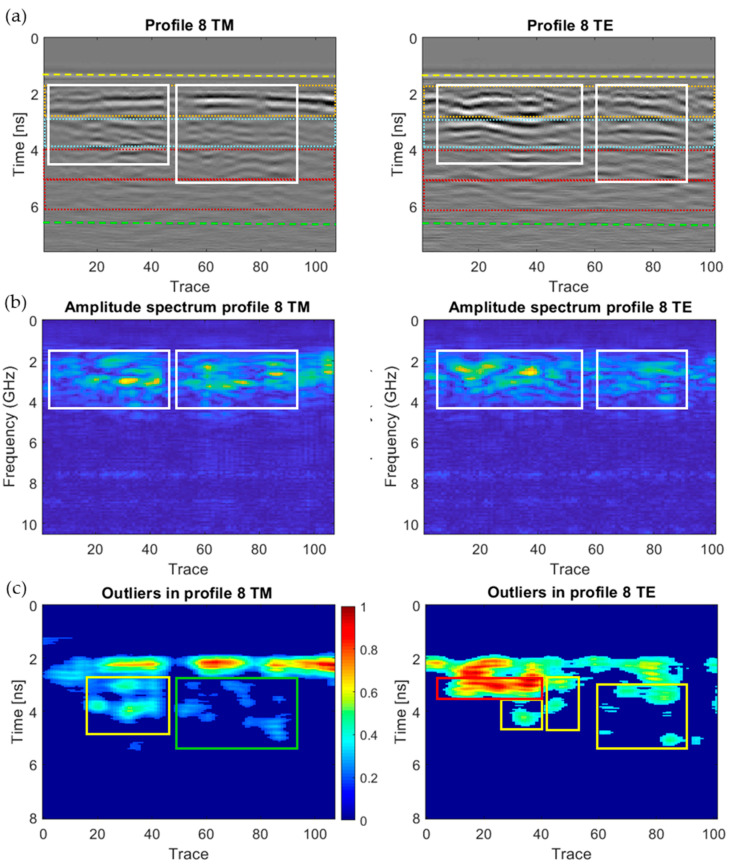
Graphics obtained for GPR profile 8 (Wall_2): (**a**) radargram (amplitudes in greyscale) (the colour code used in interpretation is the same as in Figure 12), (**b**) amplitude spectrum of the radargram (colour-scale map), and (**c**) normalised amplitude value of the outliers (same scale for both subplots).

Figure 18, Figure 19, Figure 20, Figure 21, Figure 22, Figure 23 and Figure 24 present all the GPR graphics produced for both TM and TE antenna orientations in Wall_2. All the GPR profile lines have a length of 1 m (100 traces). The areas that are interpreted as internal moisture are highlighted with white rectangles in graphics (a) and (b). In the case of graphics (c), internal moisture areas are highlighted with rectangles coloured depending on the normalised amplitude value of the outliers as follows: green (from 0.25 to 0.5), yellow (from 0.5 to 0.75), and red (from 0.75 to 1.0).

First, Figure 18 shows the GPR results produced for Profile 2, in which moisture was identified in the trace ranges from 0 to 10 and from 20 to 45 with the TM antenna orientation, and in the trace ranges from 0 to 10 and from 20 to 65 with the TE orientation. It is important to mention here that moisture was detected based on three assumptions:

1 The attenuation observed at the deeper layers of Wall_2 (Figure 18a).
  It is important to mention that moisture content causes a severe change in the dielectric properties of the materials, which leads to a decrease in the propagation velocity in the media and the subsequent signal attenuation. Observing the radargrams produced by Profile 2 (Figure 18a), the attenuation occurs at the external row of the double layer of ceramic bricks and render coat (from 4 to 6.5 ns). This interpretation is consistent with the results obtained in the boxplot (Figure 13), and it confirms that the value of the median of the 2-norm of the traces is lower in Wall_2 than in Wall_1 because of the attenuation produced by deeper moisture (there are no reflections in the coda).
2 The high-amplitude values of the reflected signals in moisture areas (Figure 18b).
  The reflection occurs when the propagating EM radar wave encounters a discontinuity in the EM properties of the media. The strength or intensity (also called amplitude) of the reflected fields is proportional to the magnitude of the dielectric constant (permittivity) change, which is characterised by the Reflection Coefficient (RC). For a non-conductive medium and perpendicular incident wave, coming from an upper medium (1) and going to a lower medium (2), RC is expressed as:
  (2) $$\mathrm{RC}=\frac{\sqrt{{Ɛ}_{1}}-\sqrt{{Ɛ}_{2}}}{\sqrt{{Ɛ}_{1}}+\sqrt{{Ɛ}_{2}}}$$
  where Ɛ_1_ is the dielectric constant of the upper medium and Ɛ_2_ is the dielectric constant of the lower medium.
  The RC increases as the dielectric contrast increases, and it takes a value between −1 ≤ RC ≤ 1. Thus, the reflection will be better identified when the RC shows a higher value and, on the contrary, the reflection may not be recorded if the coefficient is too low. For a brick/water interface, with dielectric constants of 5 and 81 for clay and fresh water [70], respectively, the RC is ±0.6. Observing the graphics in Figure 18, moisture areas (white rectangles) show more prominent reflections with higher amplitude (a) and stronger spectrum (b).
3 Looking for outliers deeper than ≈2.5 ns (the end of the interior bricklayer) (Figure 18c).
  Observing the outliers in Figure 18c, moisture areas (inside coloured rectangles) are identified from traces 0 to 10 and 20 to 60, although the highest moisture is represented as red colour (normalised amplitude value of the outliers close to 1) corresponding to the trace range from 30 to 45 (red rectangle in the TM configuration). The area having the most severe moisture interpreted is coincident with the EPS insulation boards (from 3 to 4 ns), but a low-severity moisture level is also deeply detected at the inner row of the double layer of ceramic bricks (from 4 to 5 ns).

The analysis and interpretation of the rest of the GPR profiles was performed with the same assumptions applied to Profile 2. Table 4 summarises the interpretation of the GPR graphics obtained for all the GPR profile lines measured in Wall_2 (Figure 18, Figure 19, Figure 20, Figure 21, Figure 22, Figure 23 and Figure 24) considering both the TM and TE antenna orientations. 

**Figure 25:**
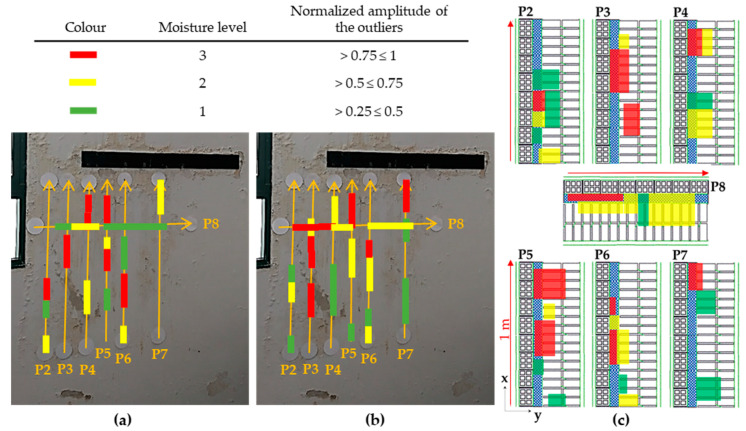
Internal moisture areas detected in Wall_2 through the analysis of the GPR profiles classified by three levels of moisture (1—low (green rectangles), 2—medium (yellow rectangles) and 3—high (red rectangles)): (**a**) TM orientation, (**b**) TE orientation, and (**c**) cross-section of the wall composition showing the depth and position of the moisture areas detected with both TM and TE configurations (red arrows indicate the profile direction).

To conclude, Figure 25 illustrates the internal moisture areas detected (projected onto a plan view map) for both the TM (a) and TE (b) configurations, through the analysis of the GPR profile lines, and characterised by three levels of moisture severity based on the normalised amplitude value of the outliers: 1—low (green rectangles), 2—medium (yellow rectangles), and 3—high (red rectangles). Additionally, Figure 25c shows the depth and position of those moisture areas detected in the interior of Wall_2 (see the wall composition and measures in Figure 11c) by levels of severity. Note that all the moisture areas detected with both TM and TE are merged in Figure 25c, and the most severe level is shown when two different levels spatially coincide. The interpretation of Figure 25 is made in the following section when combining the GPR results with the IRT results. 

### 3.3. IRT-GPR Combined Interpretation

The results previously interpreted from IRT and GPR separately are herein combined in order to analyse the complementary information provided by using these techniques together when detecting and characterising moisture areas, in this case applied on interior walls of buildings. 

Regarding detection, no moisture has been identified on Wall_1 with neither the IRT method (superficial moisture) applied on Area_1 and Area_2 (Figure 14) nor the GPR method (internal moisture) applied on Profile 1 (Figure 12). In contrast, moisture areas are detected on Wall_2 with both the IRT method applied on Area_4_1 and Area_5 (Figure 15, Figure 16 and Figure 17) and the GPR method applied from Profiles 2 to 8 (Figure 18, Figure 19, Figure 20, Figure 21, Figure 22, Figure 23 and Figure 24).

**Figure 26:**
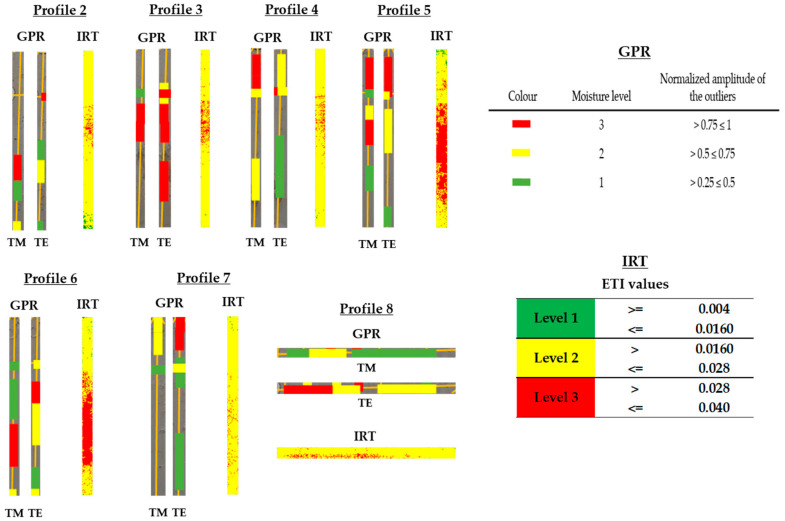
Characterisation of the most severe moisture areas by the IRT method (superficial moisture areas) and the GPR method (internal moisture areas).

Figure 26 shows the result of the interpretation of the IRT data on each of the regions of the profiles measured with the GPR antenna, with the purpose of jointly interpreting the identification of the most severe moisture areas in Wall_2. It should be noted that the processes of erosion and dilation are not applied in this case in the IRT method, because the regions of the profiles are small areas, and therefore the accuracy of the establishment of the different levels of moisture severity could be distorted. In addition, three different moisture severity levels have been applied in the IRT analysis to be consistent with the number of levels used for the GPR analysis.

From Figure 26, and with respect to the GPR results (plan view map of the internal moisture areas), the highest level of moisture severity (Level 3) is identified in the lower height of Profile 2, rising to a maximum height in Profile 4 and remaining at the top in Profile 5. Then, the highest moisture severity returns to a lower height in Profile 6 and rises again in Profile 7. As for Profile 8, horizontal, the highest moisture severity is to the left coinciding with the highest moisture in the uppermost heights observed for the vertical profiles (from Profile 3 to Profile 5). As for the lowest level of moisture severity (Level 1), it predominates in the lower height along all the vertical profiles (except for Profiles 3 and 6, in which Level 1 predominates in the upper half of the profiles). Finally, the intermediate level of moisture severity (Level 2) follows the same trend as Level 3 in all vertical profiles. In the case of the horizontal profile (Profile 8), Levels 1 and 2 are evenly distributed.

Conversely, the IRT results barely show moisture areas with the lowest level of moisture severity at the surface and close to the surface (Level 1), prevailing the intermediate severity level (Level 2). Referring to the highest severity level (Level 3), it is located approximately in the central zone from Profiles 2 to 4, with moisture decreasing in height and increasing in area in Profiles 5 and 6 but being insignificant in Profile 7. In Profile 8, the highest moisture severity is also located to the left but it is just as negligible as the result shown for Profile 7.

The lack of consistency in the moisture severity levels independently established by IRT and GPR is because IRT identifies superficial moisture, while GPR detects internal moisture. However, in those profiles where there are similarities in the severity levels identified by both techniques, it certainly means that moisture is at more superficial layers. Proof of this affirmation is the depth of the reflections/outliers highlighted in the radargrams of Profiles 6 and 8, which present the best matching in the moisture severity established by IRT and GPR. From the GPR results, it is possible to interpret that Profiles 6 and 8 present high moisture severity level at a lower depth (see Figure 25c), within the EPS insulation layer barrier; whereas Profiles 2, 4, and 7 present high moisture severity level deeper in the wall, which is extended between the EPS insulation layer and the first row of the double-layer of ceramic bricks, and Profiles 3 and 5 present a high moisture severity level at deeper layers, coinciding with the inner layer (first row) of ceramic bricks.

**Figure 27:**
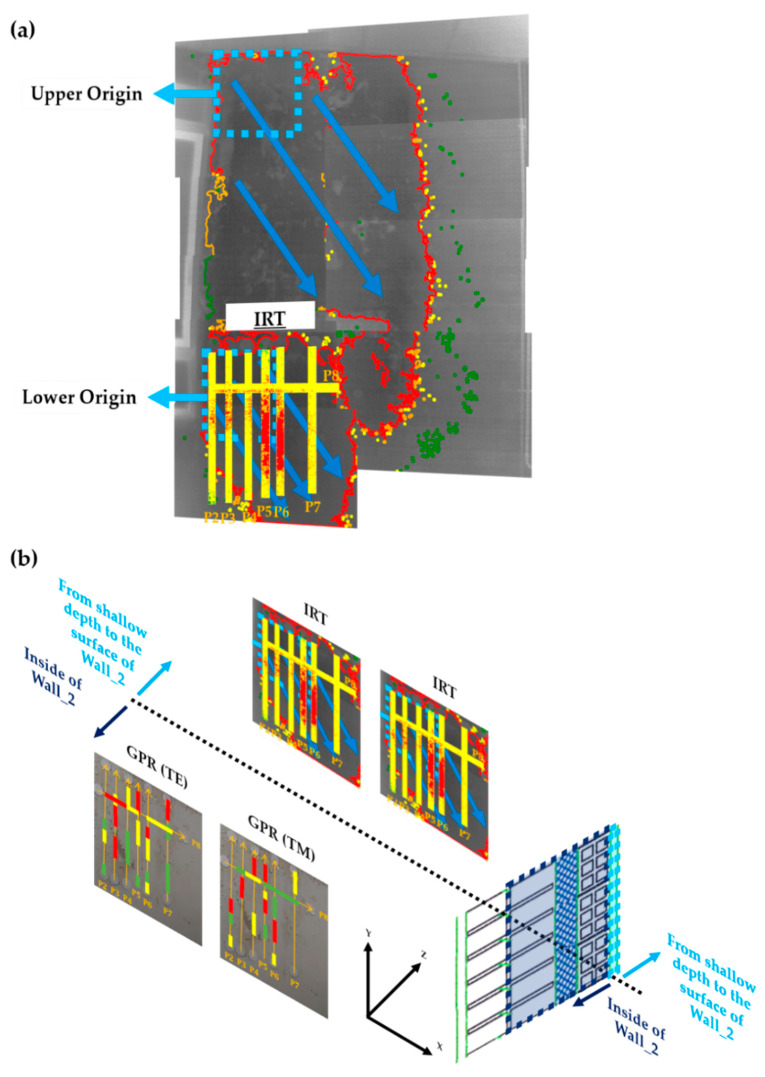
(**a**) Joint representation of the general and partial results obtained from IRT, and (**b**) illustration of both the IRT and GPR results from a lateral view aiming to show the displacement of water through Wall_2.

Figure 27 groups Figure 11, Figure 17 and Figure 26 for the joint understanding of the IRT and GPR results. Figure 27a merges the general (entire Wall_2) and the partial (regions coinciding with the GPR profiles measured through Wall_2) results obtained with the IRT survey, while Figure 27b shows a joint illustration of the results from a lateral view (spatial relationship) in order to show the displacement of water through Wall_2 through the spatial relationship of the results from IRT and GPR.

Table 5 describes the distribution of the moisture severity detected by each technique separately as well as the interpretation of the water movement achieved from the combination of techniques. 

### 3.4. Validation of IRT-GPR Interpretation

**Figure 28:**
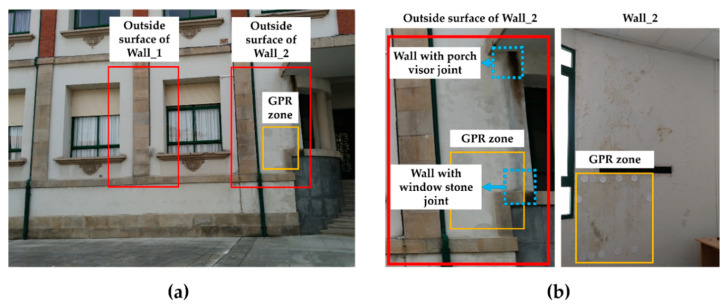
(**a**) Visible image of the outside surfaces of Wall_1 and Wall_2, and (**b**) detail of the moisture deterioration visible on both the exterior surface of Wall_2 and Wall_2. The orange rectangle highlights the region surveyed by GPR, while blue dashed line rectangles highlight the areas with the highest visible deterioration on the exterior surface of Wall_2.

A visual analysis of the exterior surface of both Wall_1 and Wall_2 is performed in this section in order to validate the conclusions about water entry and movement reached with the separate (Section 3.1 and Section 3.2) and combined (Section 3.3) interpretations of the IRT and GPR results. Thus, Figure 28a shows the visible image of the exterior surface of both walls. Analysing the exterior surface of Wall_1, no moisture damage is visible, which is consistent with the interpretations achieved from both IRT and GPR data.

However, when comparing the outside surfaces of Wall_2 and Wall_1, a certain degree of deterioration can be seen on the exterior surface of Wall_2. Analysing in more detail (Figure 28b), the detachment of the render coat on the outside surface of Wall_2 (left) is visible at an upper position than the detachment of the plaster coat on Wall_2 (right). Moreover, there are two zones with a differentiated degree of visible detachment on the outside surface of Wall_2: (i) one at the joint of the outside surface of Wall_2 with the porch visor, and (ii) another at the joint of the outside surface of Wall_2 with the window stone. These joints probably have poor insulation so that water could have accumulated and penetrated to the interior. 

Next, the combination of Figure 27 and Figure 28b allows corroborating that the visible deterioration (detachment) on the external surface of Wall_2 matches with the interpretations achieved:

1 The joints with the most visible detachment on the outside surface of Wall_2 (blue dashed line rectangles in Figure 28b) correspond to the ‘Upper Origin’ and ‘Lower Origin’ from the interior (Wall_2) with a slight difference in height (around 25 cm), being the joints above the interior detachment. Thus, the entry of water from the exterior to the interior can be deduced, which is in agreement with the porosity of the materials composing the wall, as seen in Figure 11c (from exterior to interior): ceramic bricks (porosity 33%), EPS (porosity 15%), and double-hollow bricks (porosity 45%) [71,72].
2 By analysing the distribution of the degree of visible deterioration on the external surface of Wall_2, it is possible to confirm the circulation of water downwards by gravity and to the right (seen from the interior). The existence of two movements of water from different origins, being Profile 6 the border, is also confirmed.

## 4. Conclusions

This papers presents both the separate and combined application of IRT and GPR techniques for the detection and characterisation of moisture affecting the interior surface of two walls (one without visible signs of moisture (Wall_1) and the other with signs of moisture (Wall_2)) of a building. Based on the analysis of the temperature distribution in each thermal image, the IRT allows delineating the contours of superficial moisture areas. A thermal parameter (ETI) is determined for each delineated area in order to provide an estimation of moisture severity. Regarding GPR, different algorithms based on the amplitude and spectrum of the received signals are developed for the detection and characterisation of internal moisture: (i) radargram, (ii) amplitude spectrum of the radargram, and (iii) normalised amplitude value of the radargram outliers. The latter is used to determine different levels of moisture severity. Two different antenna orientations were tested: TM and TE, showing complementary results for the detection and characterisation of internal moisture. According to this test, the use of a dual-polarised antenna is recommended for near-surface inspections. 

From the separate interpretations of IRT and GPR, it is possible to conclude that both NDT techniques have confirmed no moisture in Wall_1 (healthy wall), whereas superficial (IRT) and internal (GPR) moisture is detected in Wall_2 (visibly damaged wall). The moisture severity levels defined by each technique in Wall_2 were generally different, which is understandable due to the fact that IRT provides information from a lower range of depth (less than 1.5 cm with passive IRT) than GPR (from 1 cm up to 30 cm in the damaged Wall_2 with the 2300 MHz antenna). Other interesting findings are as follows:

- The superficial water distribution is determined from the analysis of the shape of the superficial moisture areas (delimited contours) and from the severity levels defined by IRT.
- The internal accumulation of water is estimated from the analysis of traces with stronger and prominent reflections (position and depth) in the radargrams (GPR), as well as from the severity levels defined by the normalised amplitude value of the radargram outliers.

On the other hand, the combined IRT-GPR interpretation allows estimating:

- The relative water depth inside Wall_2. Moisture areas having similar levels of moisture severity with both IRT and GPR are interpreted as having lower water depth (depth region where both techniques show some overlapping in penetration).
- The displacement of moisture between the inside and the surface of Wall_2 (comparing the most severe moisture detected with IRT and GPR). With the support of photography from the outside surface of Wall_2, it was possible to estimate that water enters from the outside through two joints, rising slightly and moving to the right (seen from interior) due to a possible higher porosity in those zones. Furthermore, it has been understood that water also moves downwards due to the effect of gravity.

Summarising, this paper makes a contribution to the complementary application of both IRT and GPR techniques for the detection and characterisation of moisture in building indoors. Then, a new inspection approach, and its benefits, are presented to the community of practice engaged with the prevention and maintenance tasks in the building sector, either for heritage or new buildings (and being valid for both in situ punctual and in situ monitoring tests as well as for laboratory tests). Further research will be the integration of both 2D and 3D IRT and GPR imaging into a Building Information Modelling (BIM). Apart from these data that allow mapping moisture, new algorithms will be developed to provide a quantitative estimation of the water content and depth of each moisture area.

## Figures and Tables

**Table 1:** Specific and common merits of InfraRed Thermography (IRT) and Ground-Penetrating Radar (GPR) in building inspection.

IRT Merits	GPR Merits	Common Merits
- Non-contact tool [17]; in contrast to GPR operating with ground-coupled antennas and distance acquisition mode, when the contact of the antenna and the survey wheel with the surface is needed (in spite of the difficulty to reach upper walls). However, these ground-coupled antennas can operate with a separation of 2–5 cm from the surface, and there are GPR antennas operating in a non-contact mode (air-coupled antennas) [47] - Ability to analyse any surface regardless of the type of material [17]; this is not possible with GPR, since metal surfaces are not penetrated by the radar waves [48]. In addition, the accuracy of GPR depth data depends on the calibration, being reduced in certain materials that allow limited GPR signal penetration [33] - IR camera can monitor the temperature of many points of a building at the same time [49]	- Low dependence on the environmental conditions [50] - - Independence of the thermal condition of the surface under study [50] - Independence of surface parameters for the correct reading, in contrast to IRT, which is dependent on the emissivity parameter [51] - Long-range depth analysis (from 1 cm to 10 m depending on the frequency) [48,52]. For example, the maximum depth in IRT is only 1.5 cm for plaster [15] and 5 cm for reinforced concrete [20] when using active IRT	- Evaluation of the properties of the building elements without causing damage [16,19] - Possibility to perform large-scale studies of materials [53,54] - Real-time operation [17,55] - Compared to alternative intrusive investigations, GPR and IRT require less time and costs, and they produce less disruption to the users of the buildings [18,34] - Repeatable and reliable tools [17,55] - High maneuverability [17,56] - Interpretation of acquired data in 2D and even 3D [28,46,57] - Location and estimation of the extension of water before permanent damage occurs [28,36,38]

**Table 2:** Specifications of the InfraRed (IR) camera used.

Model	NEC TH9260
Sensor type	Uncooled focal plane array (μbolometer)
Thermal image/pixels	640 (H) × 480 (V)
Resolution (°C)	0.1
Accuracy	±2 °C or ±2% of reading, whichever is greater
Spectral ranges (μm)	8 to14

**Table 3:** Filters and parameters used for GPR data processing.

Filtering	2300 MHz
Dewow	0.5 ns
Gain function	Lineal: 1 Exponential: 1
Subtracting average	250 traces
Migration (Kirchhoff)	Velocity: 10.0 cm/ns (summation width: 5)

**Table 4:** Interpretation of the GPR graphics obtained for each of the profile lines produced.

Profile	GPR Graphics Interpretation			
	(a)	(b)	(c)	Internal Moisture Interpretation
	Stronger Reflections in Trace-Ranges and Depth (ns):	Higher Spectrum in Trace-Ranges:	Value of Outliers: High (0.75–1), Medium (0.5–0.75) and Low (0.25–0.5)	
P2	TM = [0–10; <4.5 ns] and [20–45; <4.5 ns]; TE = [0–10; <4.5 ns] and [20–65; <4.5 ns]	TM = [0–10] and [20–45]; TE = [0–10] and [20–65]	High: TM = [30–45]. Medium: TM = [0–10]; TE = [25–45]. Low: TM = [15–30]; TE: [0–10] and [45–65]	High-severity moisture at the EPS layer with the TM mode. Areas with lower-severity level are also detected at the inner row of the ceramic bricks
P3	TM = [50–70; <4 ns]; TE = [20–40; <5 ns] and [50–80; <4 ns]	TM = [50–70]; TE = [20–40] and [50–80]	High: TM = [50–70]; TE = [20–40] and [50–70]. Medium: TM = [70–80]	High-severity moisture: TM at the EPS layer; TE extended up to the inner row of the ceramic bricks
P4	TM = [20–40; <4.5 ns] and [70–90; <4 ns]; TE = [20–50; <4.5 ns] and [65–90; <4.5 ns]	TM = [20–40] and [70–90]; TE = [65–90]	High: TM = [70–90]. Medium: TM = [20–40]; TE = [70–90]. Low: TE = [20–50]	The most significant moisture (high values of outliers) is detected at the EPS layer
P5	TM = [20–30; <4 ns], [40–60; <4.5 ns] and [75–95; <4 ns]; TE = [0–10; <5 ns], [40–60; <4 ns] and [75–95; <5 ns]	TM = [20–30], [40–60] and [75–95]; TE = [0–10], [40–60] and [70–95]	High: TM = [40–55] and [75–95]; TE = [75–95]. Medium: TM = [55–65]; TE = [35–55]. Low: TM = [20–30]; TE = [0–10]	High-severity moisture: TM at the EPS layer; TE extended up to the inner row of the ceramic bricks
P6	TM = [0–10; <4.5 ns], [30–50; <4.5 ns] and [55–75; <3.5 ns]; TE = [0–10; <5 ns], [10–25; <4.5 ns], [30–60; <4 ns] and [60–75; < 3.5 ns]	TM = [0–10], [30–50] and [55–75]; TE = [0–10] and [55–70]	High: TM = [35–50]; TE = [60–70]. Medium: TM = [0–10]; TE = [0–10] and [30–60]. Low: TM = [55–75]; TE = [10–20]	High-severity moisture at the EPS layer. TE allows also the detection of lower moisture at the inner row of ceramic bricks
P7	TM = [85–100; <4.5 ns]; TE = [10–40; <5.5 ns], [65–85; <5 ns] and [85–100; <4.5 ns]	TM = [85–100]; TE = [10–40], [65–85], and [85–100]	High: TE = [85–100]. Medium: TM = [85–100]. Low: TE = [10–35] and [65–80]	High-severity moisture at the EPS layer with the TE configuration. TE also detects low level at the inner row of ceramic bricks
P8	TM = [0–45; <4.5 ns] and [50–90; <5 ns]; TE = [10–40; <3.5 ns]; [40–55; <4.5 ns] and [60–90; <5 ns]	TM = [0–45] and [50–90]; TE = [10–55] and [60–90]	High: TE = [10–40]. Medium: TM = [15–45]; TE = [40–50] and [60–90]. Low: TM = [50–90]	High-severity moisture at the EPS layer with the TE configuration
All profiles (Wall_2)	Attenuation at deeper layers (4 to 6.5 ns)			Moisture detection not allowed at the external row of ceramic bricks and render coat (from 5.5 to 6.5 ns)

**Table 5:** IRT-GPR combined interpretation for each of the profile lines produced.

Profile	GPR Results (Internal)	IRT Results (Superficial)	IRT-GPR Interpretation
3 4 5 7	High moisture at upper zones	High moisture at the central-upper zone for Profiles 3 and 4, decreasing in height and increasing in area towards Profile 5, being insignificant in Profile 7	Water moves from the inside to the surface of Wall_2. The reason is that high moisture is detected at upper heights with GPR (internal) rather than with IRT (superficial), moving the water down by the effect of gravity (confirming the effect of gravity proposed in Section 3.1).
2	High moisture at the central-lower zone	High moisture at the central-upper zone	Considering the fact that Profile 2 is the only profile showing high moisture levels at upper heights with IRT (superficial) than with GPR (internal), the assumption of water moving from the surface to the inside of Wall_2 is discarded in this case. Then, it is proposed that the difference in height is due to a water ingress from the exterior at the ‘Lower Origin’, and with the water accumulating on the shallow surface rather than falling by gravity. Water leakage is discarded as there are no inner water supplies in Wall_2. Rising water by capillarity is also discarded due to the considerable height above the ground (the profile lines start 75 cm above the ground level) and the proper ventilation inside the room (keeping RH in a stable and adequate range).
6	High moisture at the central-lower zone	High moisture at the central-lower zone, and with the highest area together with Profile 5	Profile 6 shows high internal moisture level at shallow depths with GPR (EPS layer) and the biggest superficial moisture area with IRT (similar behavior observed for Profile 5). Considering also that Profile 7 has a severe moisture level at higher depths (from the EPS layer to the double hollow bricks), and superficial moisture is barely observed, it is proposed that Profile 6 acts as a boundary between two different movements of water. Specifically, for Profile 2 to 6, the water comes from the ‘Lower Origin’, moving from the exterior to the interior by the effect of gravity, although it also rises slightly to the bottom of the rack due to the higher degree of porosity in that zone (according to the high moisture location in Profiles 3 to 5 and Figure 27a). Observing Figure 27a, it can be seen how water also moves to the right due to the higher degree of porosity in the lower right region of Wall_2, stopping its expansion after Profile 6. With respect to Profile 7, the internal and superficial moisture can be further associated with the downward and rightward water movement from the ‘Upper Origin’ thanks to the contours delimited by IRT.
